# Loss of bone morphogenetic protein signaling in fibroblasts results in CXCL12-driven serrated polyp development

**DOI:** 10.1007/s00535-022-01928-x

**Published:** 2022-11-03

**Authors:** Sarah Ouahoud, Barbara Florien Westendorp, Philip Willen Voorneveld, Subinuer Abudukelimu, Pim Johan Koelink, Elena Pascual Garcia, Jessica Flora Isabella Buuren, Tom Jacob Harryvan, Kristiaan Jan Lenos, Tom van Wezel, Johan Arnold Offerhaus, Arantza Fariña-Sarasqueta, Stijn Crobach, Marije Slingerland, James Christopher Henry Hardwick, Lukas Jacobus Antonius Christiaan Hawinkels

**Affiliations:** 1 Department of Gastroenterology and Hepatology, Leiden University Medical Center, C4-P, P.O. Box 9600, 2300 RC Leiden, The Netherlands; 2 Tytgat Institute for Liver and Intestinal Research, Department of Gastroenterology and Hepatology, Amsterdam University Medical Center, Amsterdam, The Netherlands; 3 Laboratory for Experimental Oncology and Radiobiology, Center for Experimental and Molecular Medicine, Cancer Center Amsterdam, Amsterdam, The Netherlands; 4 Amsterdam Gastroenterology and Metabolism, Amsterdam University Medical Center, Amsterdam, The Netherlands; 5 Department of Pathology, Leiden University Medical Center, Leiden, The Netherlands; 6 Department of Pathology, University Medical Center Utrecht, Utrecht, The Netherlands; 7 Department of Pathology, Amsterdam University Medical Center, Amsterdam, The Netherlands; 8 Department of Medical Oncology, Leiden University Medical Center, Leiden, The Netherlands

**Keywords:** BMPR1A, Fibroblasts, CXCL12, Consensus molecular subtypes 4, Colorectal cancer

## Abstract

Mutations in Bone Morphogenetic Protein (BMP) Receptor *(BMPR)1A* and SMAD4 are detected in 50% of juvenile polyposis syndrome (JPS) patients, who develop stroma-rich hamartomatous polyps. The established role of stromal cells in regulating BMP activity in the intestine implies a role for stromal cells in polyp development. We used conditional Cre-LoxP mice to investigate how specific loss of BMPR1A in endothelial cells, fibroblasts, or myofibroblasts/smooth muscle cells affects intestinal homeostasis. Selective loss of BMPR1A in fibroblasts causes severe histological changes in the intestines with a significant increase in stromal cell content and epithelial cell hyperproliferation, leading to numerous serrated polyps. This phenotype suggests that crucial changes occur in the fibroblast secretome that influences polyp development. Analyses of publicly available RNA expression databases identified CXCL12 as a potential candidate. RNAscope in situ hybridization showed an evident increase of *Cxcl12*-expressing fibroblasts. In vitro*,* stimulation of fibroblasts with BMPs resulted in downregulation of *CXCL12*, while inhibition of the BMP pathway resulted in gradual upregulation of *CXCL12* over time. Moreover, neutralization of CXCL12 in vivo in the fibroblast-specific BMPR1A KO mice resulted in a significant decrease in polyp formation. Finally, in CRC patient specimens, mRNA-expression data showed that patients with high *GREMLIN1* and *CXCL12* expression had a significantly poorer overall survival. Significantly higher *GREMLIN1*, *NOGGIN,* and *CXCL12* expression were detected in the Consensus Molecular Subtype 4 (CMS4) colorectal cancers, which are thought to arise from serrated polyps. Taken together, these data imply that fibroblast-specific BMP signaling–CXCL12 interaction could have a role in the etiology of serrated polyp formation.

**Supplementary Information:**

The online version contains supplementary material available at 10.1007/s00535-022-01928-x.

## Introduction

Juvenile Polyposis Syndrome (JPS) is a rare autosomal-dominant hereditary disorder in which patients form numerous hamartomatous polyps throughout their gastrointestinal tract, which gives them an increased lifetime risk of developing gastrointestinal cancer [1]. Germline mutations in two genes, *Bone Morphogenetic Protein Receptor 1A* (*BMPR1A),* and *SMAD4* have been identified to cause JPS in 50–60% of the patients [2]. It is unclear how these mutations might contribute to polyp development, but it is conspicuous that both genes encode for crucial signaling proteins of the BMP pathway [3].

The BMPs are a group of growth factors that belong to the transforming growth factor (TGF)-β family. These growth factors signal through the type 1 (e.g., BMPR1A and BMPR1B) and type 2 (BMPR-II) receptors upon which SMAD1/5/8 molecules are phosphorylated. Subsequent complex formation with SMAD4 results in transcription of BMP target genes [4]. Since BMP target genes are involved in numerous biological processes, this pathway is considered to be crucial for maintaining adult intestinal tissue homeostasis [5, 6]. In the intestine, BMPs promote epithelial cell differentiation while restricting their proliferation. BMP antagonists, such as Gremlin1 and Noggin, are crucial when it comes to regulating the BMP signaling amplitude in the crypt base. There, the BMP antagonists bind BMPs to prevent BMP-mediated cell differentiation, ensuring the preservation of stemness. It is currently thought that tissue homeostasis is dependent on interactions between epithelial cells and the stroma [7]. Especially pericryptal stromal cells, such as the fibroblasts and myofibroblasts, are thought to regulate the activity of the BMP pathway through producing ligands and inhibitors of the BMP pathway [7].

The mutations in *BMPR1A* and *SMAD4* found in JPS suggest that the BMP pathway is implicated in the development of intestinal polyps. Studies from others indicated that abrogation of intestinal BMP signaling in the epithelium alone was not sufficient to trigger polyp development [8]. Since stromal cells are key players in regulating intestinal BMP signaling and JPS polyps have a stroma rich, hamartomatous phenotype it is likely that the stroma is involved in polyp formation. However, it remains unclear precisely which resident stromal cell type can contribute to polyp development.

In this study we investigated how the inactivation of BMPR1A in different resident stromal subsets affects intestinal homeostasis and polyp formation. We used several cell-specific conditional BMPR1A knockout (KO) mouse strains to determine which resident stromal cells (i.e., fibroblasts, myofibroblasts, or endothelial cells) are crucial in maintaining intestinal epithelial cell homeostasis. We show that loss of BMPR1A signaling in fibroblasts leads to polyp development through upregulation of *CXCL12*, driving epithelial cell proliferation and serrated polyp formation.

## Methods

### Animal experiments

All animal experiments performed were approved by the animal welfare committee of the Leiden University Medical Center (LUMC) and conducted according to the Dutch Experiments on Animals Act and the “Guidelines on the protection of experimental animals” from the European council. BMPR1Atm2.1Bhr (The Jackson Laboratory, Bar Harbor, Maine, USA) mice were crossbred with Tg(Cdh5-Cre/ERT2)CIVE23Mlia (endothelial cell-specific Cre), Tg(Col1a2-Cre/ERT,-ALPP)7Cpd/J (fibroblast-specific Cre), B6.Cg-Tg(Tagln-Cre)1Her/J (myofibroblast/smooth muscle cell-specific Cre) mice and Gt(ROSA26)Sortm1(EYFP)Cos (reporter mouse strain, all The Jackson Laboratory) to produce BMPR1A^fl/fl^; Rosa26-EYFP; VeCad-Cre/ERT (BMPR1A^fl/fl−VeCad^), BMPR1A^fl/fl^; Rosa26-EYFP; Col1a2-CreERT (BMPR1A^fl/fl−Col1a2^) and BMPR1A^fl/fl^; Rosa26-EYFP; SM22-Cre/ERT2 (BMPR1A^fl/fl−SM22^) mice respectively.

In the *BMPR1A-*floxed mice exon 2 of the *BMPR1A* gene is flanked with LoxP sites. Cre-mediated recombination of these LoxP sites causes a frameshift resulting in a non-functional receptor [9]. Mice were induced with 100 mg/kg tamoxifen (Merck, Darmstadt, Germany) dissolved in sunflower oil (20 g/l) and injected intraperitoneally once a day for three consecutive days.

Cre-negative littermates injected with tamoxifen, Cre-positive littermates injected with sunflower oil, or BMPR1A^wt/wt^ mice injected with tamoxifen were used as control mice. Induced mice will be referred to as “BMPR1A^Δ^”, while controls will be referred to as “BMPR1A^ctrl^”. Mice were sacrificed 90, 180, or 365 days post-induction. Both the small and large intestines were opened longitudinally, cleaned of feces, rinsed with ice-cold phosphate-buffered saline (PBS), and checked for the presence of polyps. Subsequently, intestines were fixed for 24 h in 4% paraformaldehyde and processed for histological analysis. Paraffin blocks with intestinal tissue of CXCL12/GFP knock-in mice were a kind gift from Prof. dr. Nagasawa and Prof. dr. Scamble [10].

For the treatment of mice with FK-506 (Merck) or LIT-927(Selleckchem, Huissen, The Netherlands), BMPR1A^fl/fl−Col1a2^ mice were induced as described. Starting from week 1, mice received 2 intraperitoneal injections per week with 1 mg/kg FK-506 dissolved in 10% DMSO and sterile water or with 25 mg/kg LIT-927 dissolved in 5% DMSO, 40% PEG300, and 1% Tween80 in sterile water or solvent controls. Mice were sacrificed 90 days post-induction. All in vivo experiments were repeated at least 2 times except for the treatment experiments.

### Cell culture and in vitro stimulations

The colonic fibroblast cell line CCD-18co was purchased from ATCC (American Type Culture Collection, Manassas, VA). 18co cells were cultured in DMEM/F12 GlutaMax (Gibco, Thermo Fisher Scientific, Bleiswijk, The Netherlands). Cell growth medium was supplemented with penicillin (100 U/ml, Gibco), streptomycin (100 µg/ml, Gibco), and 10% FCS (Gibco) unless stated otherwise. Fibroblasts were seeded in six well plates and stimulated for up to 96 h with 100 ng recombinant BMP2 (R&D Systems, Abingdon, UK), 200 nM LDN-193189 (Merck), or 100 ng recombinant Noggin (Peprotech, Rocky Hill, NJ, USA) in DMEM/F12. Normal intestinal mouse organoids were stimulated with 50 ng recombinant CXCL12 (Biolegend, Uithoorn, The Netherlands) and monitored for 5 consecutive days.

### Transwell migration and invasion assay

For transwell migration assays, 50,000 CCD-18co fibroblasts together or without addition 200 nM of the BMP inhibitor LDN-193189 were added to the upper chamber of 8.0-μm pore size ThinCert (Greiner Bio-One) in 0.5% FCS/ DMEM-F12. The lower compartment of the transwell system contained 10% FCS/ DMEM-F12. For invasion assays only, transwells were pre-coated with 200 µg/ml rat tail collagen type I (Ibidi, Martinsried, Germany). After 24 h, cells that had migrated or invaded through the transwell were fixed, stained with DAPI and quantified with the Cytation 5 Cell imaging Multimode Reader (BioTek instruments, Bad Friedrichshall, Germany).

### Fluorescence-activated cell sorting

Intestines from wild-type mice were opened longitudinally, cleaned of feces, rinsed with PBS, and cut into 5 mm pieces. The tissue was washed 3 times with ice-cold PBS and incubated for 20 min in 2% Fetal Calf Serum (FCS) and 5 mM Ethylenediaminetetraacetic acid (EDTA) in Hank’s Balanced Salt Solution (HBSS, Merck) under low agitation (220 rpm). Tissue was washed 3–4 times with ice-cold PBS, and the dissociated epithelium was harvested. Epithelium-free tissue was then further digested in a digestion mix consisting of 125 µg/ml Liberase (Roche, Basel, Switzerland), 40 µg/ml DNAse (Roche) and 2% FCS in HBSS. 10% FCS/ DMEM was added to stop the digestion. Samples were centrifuged for 5 min at 1200 rpm at 4 °C and washed with 20 ml ice-cold PBS. The digested tissue was passed through a 100 µm mesh filter. The single cell-suspensions were stained for 30 min on ice with an antibody mix for EpCam, CD45, CD31, and gp38 (Supplementary Table 1). 10.000–50.000 viable cells were sorted using the BD FACS ARIA III sorter (BD Bioscience, Heidelberg, Germany) for RNA isolation and mRNA-expression analysis.

### Histological analysis

Formalin-fixed and paraffin embedded (FFPE) tissues were sectioned in 4 µm sections and stained with hematoxylin and eosin. Sequential sections of the intestines were analyzed under the microscope for the presence of polyps. Alcian blue staining was performed by incubation for 15 min in a 1% alcian blue (Merck) and 3% acetic acid solution. The slides were counterstained for 1 min with neutral red (Merck) stain. The percentage of alcian blue area was determined with ImageJ software (https://imagej.nih.gov/ij/).

Immunohistochemical stainings were performed by deparaffinizing tissue, after which slides were incubated in 0.3% H_2_O_2_ in methanol to block endogenous peroxidase activity. Slides were then rehydrated, after which antigen retrieval was performed by boiling for 10 min in 10 mM sodium citrate (pH 6.0). Slides were incubated overnight with the primary antibody diluted in 1% bovine serum albumin (BSA) in PBS (Supplementary table 1). The next day, slides were washed and incubated with a biotinylated secondary antibody (1/200 in 1% BSA in PBS, Dako, Glostrup, Denmark) for 30 min. The VECTASTAIN^®^ Elite ABC-HRP kit (VECTOR Laboratories, Burlingame, CA) was used to amplify the signal while the DAB + liquid kit (Dako, Glostrup, Denmark) was used to detect HRP activity. Nuclei in the tissue were counterstained with hematoxylin (Merck). For the quantification, 5 pictures per mouse were obtained using an Olympus BX52 microscope. The Olfm4 and Ki67 staining were analyzed by quantifying the number of DAB positive cells per crypt. For the cleaved caspase 3 stainings, intestines were scored for the percentage if DAB positive cells according to the following scoring system: score 0: no staining, score 1: ≤ 10% DAB positive epithelial cells, score 2: 11–25% DAB positive epithelial cells and score 3: 26–50% DAB positive epithelial cells (Supplementary Fig. 4I). Five crypts were analyzed in each picture. Vimentin and CD45 stainings were quantified by determining the DAB positive area with ImageJ.

For immunofluorescent staining, sections were deparaffinized in xylene and rehydrated. Antigen retrieval was performed by boiling sections for 10 min in sodium citrate (pH 6), after which slides were blocked in PBT (PBS with 1% BSA and 0.1% Triton X-100 (Sigma -Aldrich) for 30 min. Slides were incubated overnight with primary antibodies. The next day, after washing in PBS, tissue sections were stained at room temperature (RT) with secondary antibodies labeled with a fluorescent dye (1:500, Alexa Fluor 488 or 546 or 594, and 647 from Invitrogen). Slides were washed in PBS and mounted with ProLong Gold Antifade reagent with DAPI (Thermo Fisher Scientific).

### RNAscope—in situ hybridization

RNAscope- in situ hybridization was performed according to the “Formalin-Fixed Paraffin-Embedded (FFPE) Sample Preparation and Pretreatment for RNAscope 2.5 assay” and “RNAscope 2.5 HD Detection Reagent—RED” protocol as provided by the manufacturer (Advanced Cell Diagnostics, ACD). In brief, for mouse tissue, 4 µm‐thick paraffin‐embedded sections were baked for 60 min at 60 °C, deparaffinized, and then boiled at 98–102 °C with the pretreatment reagent for 15 min. Protease digestion was carried out at 40 °C for 30 min, followed by the Cxcl12 (422,711; ACD) probe hybridization for 2 h at 40 °C with the target probes. Hybridized signals were amplified and visualized with Fast RED reagent. For human tissue, the pretreatment reagent step was extended to 30 min, the protease digestion was extended to 45 min, and the human CXCL12 (422,991; ACD) probe was used. After probes were detected with Fast RED, slides were washed three times with PBS followed by the staining with primary antibodies overnight at 4 °C. After washing in PBS, tissue sections were stained at RT with a secondary antibody (1:500, Alexa Fluor 488, Invitrogen) for 30 min. Slides were washed in PBS and mounted with ProLong Gold Antifade reagent with DAPI (Thermo Fisher Scientific).

### TGF-β and gremlin ELISA

The levels of total TGF-β were measured using a TGF-β1 duo-set (DY240) as previously described [11] while gremlin levels were measured with the gremlin duo-set (DY956) and a substrate reagent pack (DY999) according to the manufacturer’s instructions (R&D Systems Europe, Abingdon, UK).

### Organoid co-cultures

Normal mouse intestinal organoids were co-cultured with fibroblasts to investigate the effect of fibroblasts on epithelial cell growth. BMPR1A^∆−Col1a2^ mice were induced as indicated above and sacrificed one week after induction. Intestines were opened longitudinally, cleaned of feces, rinsed with ice-cold PBS, and sectioned in ~ 1–3 mm pieces using a sterile scalpel. The tissue was placed in a 15 ml conical tube with 4 ml digestion medium, consisting of 2.5 mg/ml dispase II (Roche), 7.5 mg/ml collagenase (Merck) in PBS, and incubated for 1.5 h at 37 °C. Hereafter, DMEM/F12 supplemented with 10% FCS was added to the tissue, and the tube was centrifuged for 5 min at 1200 rpm at 4 °C. The supernatant was discarded, and the digested tissue was cultured until fibroblast outgrowth was observed.

Intestinal organoids were obtained from wild-type mice with a C57BL/6 J background. Intestines were opened longitudinally, washed with ice-cold PBS, cut into small pieces of 2–4 mm, and transferred to a 50 ml tube. Tissue pieces were washed with cold PBS after which tissue was incubated in 5 mM EDTA (Gibco) in PBS for 30 min while constantly shaking. After removal of the supernatant, 10 ml of ice-cold 10% FCS/PBS was added and the suspension was vigorously suspended using a 10 ml pipet. The supernatant was then passed through a 70 mm cell strainer (BD Bioscience), transferred to a 15 ml tube and centrifuged at 800 rpm for 5 min. The supernatant was removed and the crypts were taken up in growth factor reduced (GFR) Matrigel (Corning Inc., NY, USA) and seeded in drops of 35 µl. The crypts were cultured in incubator at 37 °C. After 15 min, organoid medium supplemented with growth factors EGF, Noggin and R-spondin (ENR medium) (Supplementary table 2) was added to the well. Medium was refreshed every 2–3 days. Crypts were passaged every 7 days [12]. For the co-cultures, organoids were mixed 1:1 with fibroblasts in GFR Matrigel and distributed in 35 µl/ drop over a 48-well plate. Fibroblasts isolated from BMPR1A^ctrl−Col1a2^ mice were used as a control. Organoid (co-) cultures were cultured in ENR medium and monitored for 5 consecutive days.

### Transcriptional analysis

mRNA was isolated from the cells using the NucleoSpin RNA isolation kit (Macherey–Nagel, Düren, Germany) according to the manual. RNA concentrations were determined with the Nanodrop (Thermo Fisher Scientific). cDNA was synthesized from RNA using the RevertAid RT Reverse Transcription Kit (Thermo Fisher Scientific). Real-time-qPCR analysis was performed with 5 µl iQ™ SYBR^®^ Green Supermix (BioRad, Lunteren, The Netherlands), 2 µl nuclease-free water, and 0.5 µl of 10 µM gene-specific forward and reverse primers (Supplementary table 3) per reaction. Expression was normalized to β-actin according to the 2^-ddCT^ method.

For the *Cxcl12* expression in different cell populations, mRNA from sorted cells was isolated using Agencourt RNAdvance Cell v2 kit (Beckman Coulter) according to the manufacturer’s protocol thereby including a DNA digestion step. For cDNA synthesis, equal concentrations of RNA were reverse transcribed using oligo(dT), random hexamers, and M-MulV reverse transciptase (RT) from the First Strand cDNA Synthesis kit (Thermo Fisher Scientific) according to the manufacturer’s protocol. Quantitative RT-qPCR was performed using the sensifast SYBR No-ROX kit (Bioline) on a BioRad iCycler. All primer sets were intron spanning and primer specificity was tested using melting curve analyses. GeNorm was used to select multiple stable housekeeping genes. Analysis was performed by using the LinReg method [13].

### Detection of somatic mutations in BMPR1A^∆^ polyps

Polyps (*n* = 16) were identified on histopathology slides and harvested from paraffin blocks using a manual tissue microarray punch (Beecher Instruments, Inc., Sun Prairie, WI). Prior to DNA isolation, the formalin fixed paraffin embedded (FFPE) tissue was deparaffinized in xylene and washed in 70% ethanol. Genomic DNA was isolated using the NucleoSpin Tissue Genomic DNA Purification kit (Machery-Nagel, Düren, Germany) according to the manufacturer’s instructions. A custom AmpliSeq™ panel was designed using Ion AmpliSeq™ designer software (www.ampliseq.com, Thermo Fisher Scientific) to target the coding DNA sequence of the regions of interest (Supplementary table 4). Libraries were prepared with 20 ng of genomic DNA, and each sample was uniquely barcoded using molecular barcodes (Life Technologies) as reported previously [14]. Samples were pooled in equimolar concentrations and loaded on a proton chip using the IonChef (Thermo Fisher Scientific) according to manufacturer’s protocol. The samples were amplified on the chip using emulsion PCR. The chips were loaded in the IonTorrent Ion Proton sequencer (Thermo Fisher Scientific) and the pools were sequenced according to manufacturer’s protocol. DNA from an APCmin mouse and the cell lines MC38 and CT26 were included as positive controls, while DNA from a WT mouse was included as a negative control.

### Data availability

The following human *CXCL12*, *GREMLIN1,* and *NOGGIN* expression datasets were referenced during our study: human *CXCL12*, *GREMLIN1,* and *NOGGIN* expression with survival data (R2 internal identifier: ps_avgpres_tgcacolon174_agg4502a073). Expression profiles of cell populations purified from human CRC (GSE39395 and GSE39396). Gene expression of the publicly available TCGA primary colon cancer (COAD) dataset [15] and above mentioned data sets were analyzed using the R2: Genomics Analysis and Visualization Platform (http://r2.amc.nl/). A subset containing survival data was used to analyze overall survival. *GREMLIN1*, *NOGGIN,* and *CXCL12* subgroups (high or low) were identified by the R2 platform. CMS labels of TCGA COAD samples were previously reported by Guinney et al. [16]. GREMLIN or *NOGGIN* low and high expressing CMS4 subgroups were formed based on the mean expression of the complete CMS4 subgroup.

### Statistical analysis

Statistical analyses were performed with GraphPad Prism (version 8.4.2, GraphPad Software). A student’s *t*-test or one-way ANOVA was used for the calculation of *P* values. Nonparametric variants of the students *t*-test or one-way ANOVA were used when data did not show normal distribution. A *P* value < 0.05 was considered statistically significant. Statistical analyses of publicly available expression data sets were corrected for multiple testing.

## Results

### Loss of BMPR1A mediated signaling in fibroblasts, but not endothelial cells leads to the formation of serrated intestinal polyps

To investigate if loss of BMP signaling in resident stromal cells could contribute to polyp development, three different cell-specific conditional Cre-LoxP knockout mouse strains were generated. The VeCad-Cre driven model (BMPR1A^fl/fl−VeCad^) was used to investigate the loss of BMPR1A signaling specifically in the endothelial cells. A Col1a2 driven Cre (BMPR1A^fl/fl−Col1a2^) was used to investigate the effects of loss of BMP signaling in fibroblasts, while an SM22 driven Cre (BMPR1A^fl/fl−SM22^) was used to investigate the effect of BMPR1A loss in smooth muscle cells and myofibroblasts.

Mice with endothelial-specific BMPR1A loss were sacrificed 90, 180 or 356 days post-induction, after which the intestines were examined. Loss of BMPR1A signaling in endothelial cells did not result in any histological abnormalities. Immunohistochemical staining for the EYFP reporter protein showed the presence of EYFP-positive cells constituting the blood vessels (Supplementary Fig.1), confirming successful Cre-mediated recombination. Since no histological changes in the intestine were observed, this suggests that endothelial BMP signaling does not affect intestinal homeostasis significantly.

**Fig. 1:**
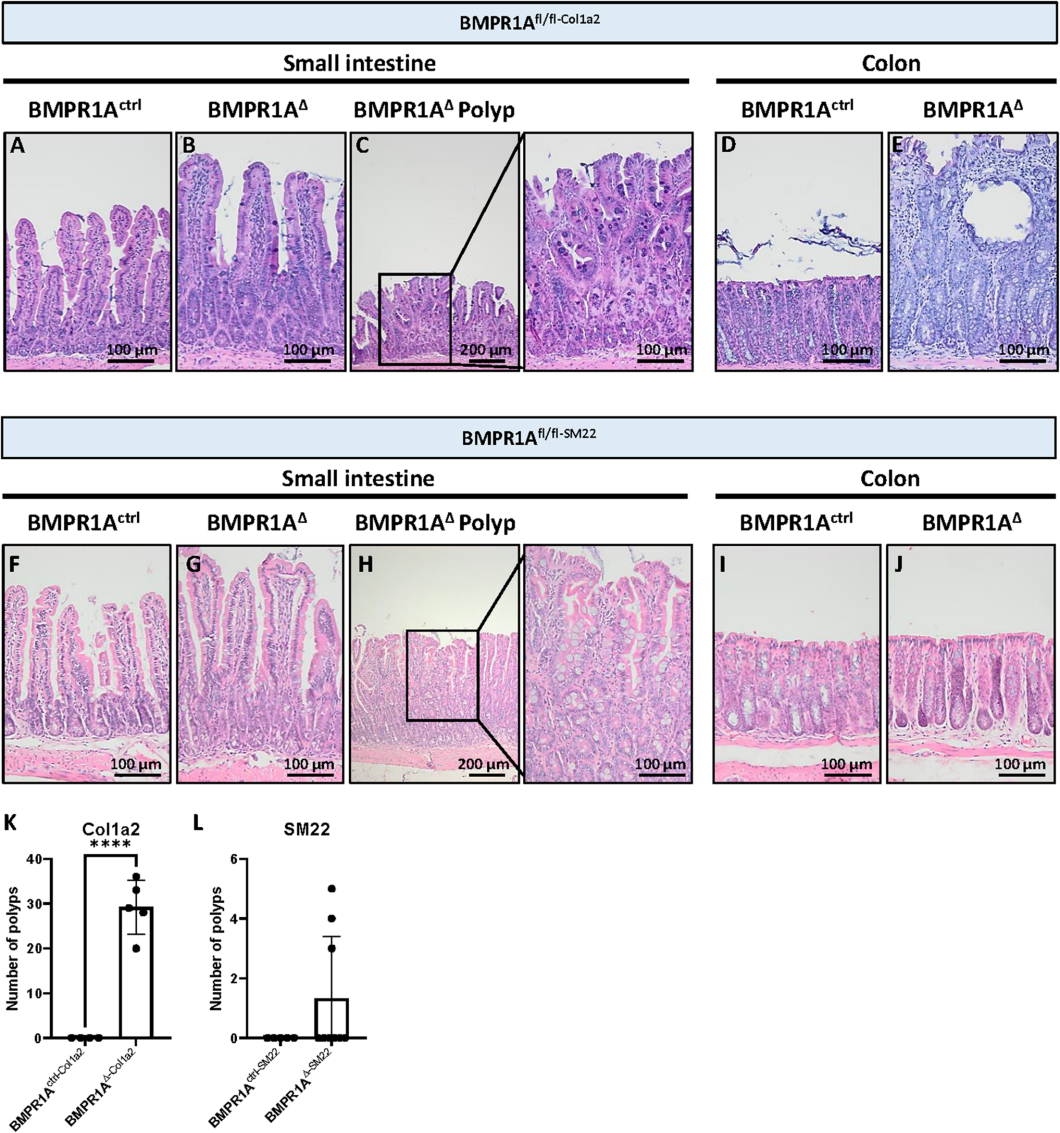
Loss of the BMPR1A in fibroblasts and myofibroblasts leads to serrated polyp formation. **A** The loss of BMPR1A signaling in *Col1a2* expressing fibroblasts results in substantial histological changes. **A**–**C** The villi appeared to be longer and wider and numerous polyps could be observed throughout the small intestine. **D**–**E** In the colon, substantial hyperproliferation could be observed and the crypt length was found to be increased 3.6-fold. Numerous cysts were observed scattered throughout the mucosa. **F** BMPR1A^∆−Col1a2^ mice formed on average 29 polyps/mouse (SD = 6). **G**–**I** On average 1 polyp developed per mouse in the BMPR1A^∆−SM22^ mice (SD = 2). D) Bars represent mean ± SD. *P*  < 0.0001 (****)

In contrast, major histological changes were observed in the intestines of BMPR1A^Δ−Col1a2^ mice 90 days post-induction (Fig. 1A–E). Immunohistochemical staining for the EYFP reporter protein showed the presence of EYFP-positive cells throughout the lamina propria, myofibroblasts and fibroblasts, of both the small intestine and colon verified successful Cre-induction (Supplementary Fig. 2A–E). Although the histological typical crypt-villus architecture was well preserved in the small intestine, numerous polyps had developed throughout the intestines, with an average of 29 polyps per mouse (Fig. 1F, *P* ≤ 0.0001). Even stronger histological changes were observed in the colon as almost the entire colonic epithelium showed signs of hyperproliferation (Fig. 1D and E). The crypt length was found to be 3.6-fold (*P* ≤ 0.0001) longer compared to the colonic mucosa of BMPR1A^ctrl−Col1a2^ mice (Supplementary Fig. 3A). Large cystic structures filled with mucous were found scattered throughout the mucosa, surrounded by elongated crypts. The diffuse pattern of epithelial hyperproliferation made it difficult to distinguish individual lesions and hampered accurate polyp quantification. The *Col1a2* promoter activity could explain the considerably stronger phenotype observed in the colon as *Col1a2* expression was fourfold higher in the colon than in the small intestine (Supplementary Fig. 3B). In addition, colon tissue appears to have more EYFP-positive cells (Supplementary Fig. 3C–G), indicating increased recombination. Next, the BMPR1A^fl/fl−SM22^ mouse strain was used to investigate if a myofibroblast-specific BMPR1A knockout could also drive the phenotype observed in BMPR1A^Δ−Col1a2^ mice. The EYFP-staining showed the presence of EYFP-positive myofibroblasts in the submucosa and EYFP-positive smooth muscle cells in the muscle tissue verifying successful Cre-induction (Supplementary Fig. 2F–J). Although polyp formation was observed in the small intestines, a less severe phenotype was seen as only 3 out of 10 mice were found to have developed intestinal polyps (Fig. 1G–L). While BMPR1A^Δ−Col1a2^ affected the colon, myofibroblast-specific knockout did not lead to histological changes in the colon.

Next, we analyzed the polyp histological subtype in the BMPR1A^Δ−Col1a2^ and BMPR1A^Δ−SM22^ mice. Contrary to what was expected, the polyps found in the BMPR1A KO mice did not resemble hamartomatous polyps, as observed in JPS patients. Instead, the polyps showed sawtooth-like serrations of the epithelium with abundant goblet cells (Supplementary Fig. 4 and 5). These features, typical for serrated polyps, are found in the polyps of both the fibroblast-specific BMPR1A knockout mice (BMPR1A^Δ−Col1a2^) and the myofibroblast-specific BMPR1A knockout mice (BMPR1A^Δ−SM22^). The presence of serrated polyps was confirmed by three independent pathologists.

Taken together, these data suggest that abrogation of fibroblast or myofibroblast-specific BMPR1A signaling, in contrast to endothelial BMPR1A signaling, leads to disturbed intestinal homeostasis and serrated polyp formation.

### Intestinal BMPR1A-expressing fibroblasts regulate epithelial cell proliferation

**Fig. 2:**
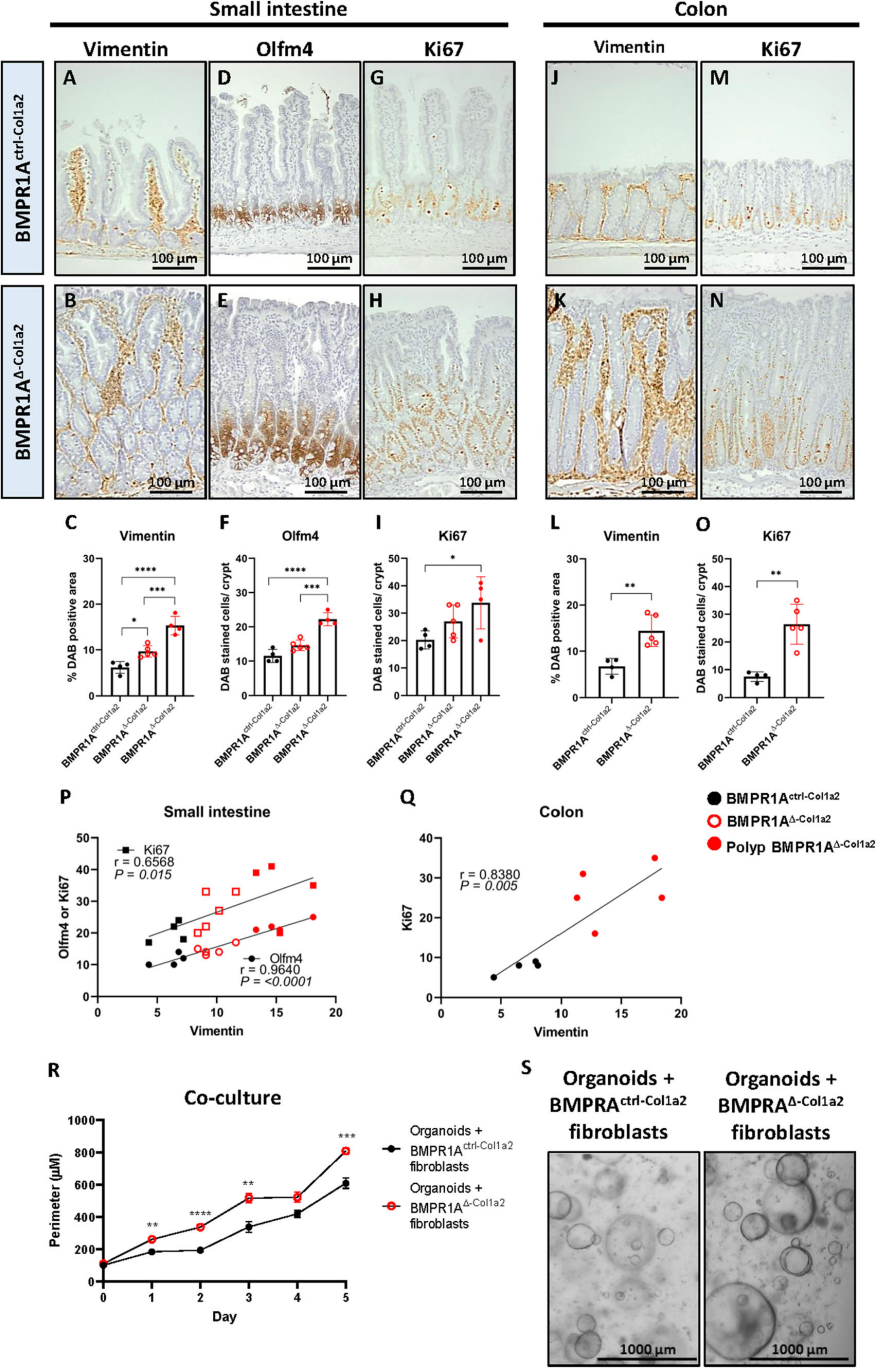
BMPR1A^Δ^-fibroblasts support epithelial cell proliferation. In the small intestines, immunohistochemical stainings on BMPR1A^∆−Col1a2^ tissue showed a significant increase of vimentin-positive stromal cells (**A**–**C**), which appeared to correlate with the increase in Olfm4^+^ stem cells (**D**–**F**) and Ki67^+^ proliferating cells (**G**–**I**). A significant increase of vimentin and Ki67 was also observed in the colon of the mice (**J**–**O**). **P**–**Q** The Pearson R correlation analyses showed a significant correlation between Olfm4 or Ki67 with vimentin in the small intestine and Ki67 and vimentin in the colon. **R**, **S** Co-culture experiments of normal intestinal organoids with either BMPR1A^ctrl−Col1a2^ fibroblasts or BMPR1A^Δ−Col1a2^ fibroblasts showed that the size of the organoids increased significantly when they were co-cultured with BMPR1A^Δ^-fibroblasts. Bars represent mean ± SD except for P in which bars represent mean ± SEM. *P* < 0.05 (*), < 0.01 (**), < 0.001 (***) and < 0.0001 (****)

To further investigate the severe histological changes observed in the intestines after disrupting fibroblast-specific BMPR1A signaling, we performed immunohistochemical stainings to investigate changes in the cellular composition. Staining for the stromal marker vimentin showed that the stroma had significantly expanded in the small intestines, polyps and colon of BMPR1A^Δ−Col1a2^ mice compared to control mice (Fig. 2A–C and J–L). Interestingly, the stromal abundance as quantified with the vimentin staining appears to correlate with the number of cells positive for the stem cell marker Olfm4^+^ and ki67^+^ proliferating cells (Fig. 2D–I and P). Olfm4 is not expressed in the mouse colon, but a similar correlation could be observed in the colon between vimentin and Ki67 (Fig. 2M–O and Q). An IHC staining for cleaved caspase 3 was performed to investigate if the increase in the number of epithelial cells could be due to a decrease in the number of cells undergoing apoptosis. An increase in the number of apoptotic cells was observed in the intestines from BMPR1A^Δ−Col1a2^ compared to controls, which was significant in the colon (Supplementary Fig. 4 J–K). To investigate whether the increase in proliferation is caused by the BMPR1A^Δ^ fibroblasts, primary fibroblasts were isolated from induced BMPR1A^Δ−Col1a2^ mice and co-cultured with normal intestinal mouse organoids. BMPR1A^Δ^ fibroblasts significantly enhanced organoid growth compared to fibroblasts isolated from Cre-negative or non-induced or non-induced BMPR1A^fl/fl−Col1a2^ mice (Fig. 2R–S), indicating a possible paracrine interaction. These data show that intestinal BMPR1A^Δ^ fibroblasts can stimulate epithelial proliferation, potentially explaining the increased proliferation observed in vivo in BMPR1A^Δ−Col1a2^ mice.

### Loss of BMPR1A in fibroblasts results in increased pERK levels and increased TGF-β activity

**Fig. 3:**
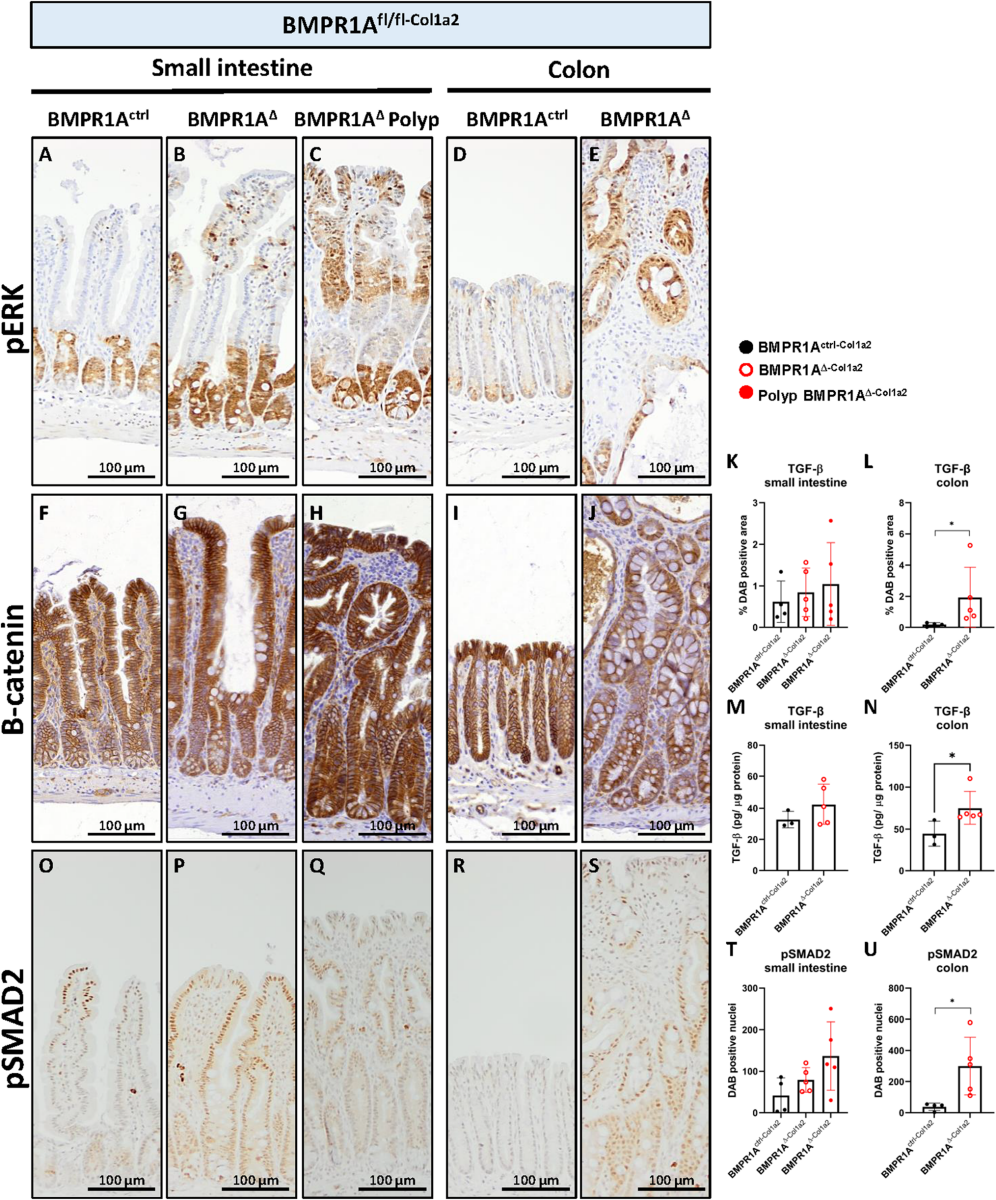
Loss of BMPR1A signaling in fibroblasts results in increased pERK levels and increased TGF-β activity. **A**–**E** pERK staining in the intestines of BMPR1A^ctrl−Col1a2^ is only observed in the (lower) crypt part of the small and large intestines. However, pERK positive cells were present at the luminal side of the intestines from BMPR1A^∆−Col1a2^mice. **F**–**J** Despite the increased membranous and cytoplasmatic B-catenin staining in the epithelial cells within the polyp, no increased nuclear localization was observed in tissue from BMPR1A^∆−Col1a2^mice compared to control. **K**, **L** Quantification of an immunohistochemical staining for TGF-β showed significant increased TGF-β in the colon of BMPR1A^∆^.^−Col1a2^mice. **M**, **N** An ELISA for TGF-β confirmed the increased TGF-β observed in the IHC staining. **O**–**U** The staining for pSMAD2 verified increased TGF-β signaling.) Bars represent mean ± SD. *P* < 0.05 (*)

CRC is thought to develop through two main independent pathways. 20–30% of CRC is thought to develop through the serrated pathway, which is associated with increased MAPK/ERK pathway activity and BRAF mutations while the majority, 70–80%, is thought to develop through the adenoma pathway [17]. To investigate if MAPK-ERK signaling was also increased in the BMPR1A^Δ−Col1a2^ mice, a staining was performed for phosphorylated ERK (pERK). While pERK positive cells were only found to be present in the (lower) crypt region of the intestines from BMPR1A^ctrl−Col1a2^, positive cells were also found to be present in the top of the villi, polyps, and colonic glands at the luminal side of the colon from BMPR1A^Δ−Col1a2^ mice (Fig. 3A–E). Next, an IHC staining for B-catenin was performed to investigate changes in Wnt pathway activity. Increased cytoplasmic and membranous staining was observed specifically in the polyps but no increased nuclear translocation was observed in both the small intestine and colon of the BMPR1^AΔ−Col1a2^ mice (Fig. 3F–J).

Since the abrogation of BMP signaling in fibroblasts could augment a number of actions in fibroblasts, we performed additional stainings to assess TGF-β signaling. TGF-β is known to be an important growth factor secreted by fibroblasts that regulates fibroblast activation and phenotypic transition into myofibroblasts [18]. Increased TGF-β staining was observed in the intestines of BMPR1A^Δ−Col1a2^ mice compared to BMPR1A^ctrl−Col1a2^ mice. However, this increase was only found to be significant in the colon of the BMPR1A^Δ−Col1a2^ mice (Fig. 3K–L and Supplementary Fig. 6A–E). Next, a TGF-β ELISA on whole tissue homogenates was performed and confirmed the increased levels of TGF-β (Fig. 3M, N). Given the fact that TGF-β is secreted as a latent molecule, we performed an additional IHC staining for phosphorylated SMAD2 (pSMAD2), a protein activated upon TGF-β signaling. pSMAD2 staining confirmed increased TGF-β activation in BMPR1A^Δ−Col1a2^ mice (Fig. 3O–U). Increased TGF- β activation did not result in an increased number of myofibroblasts as no differences in α-SMA expression were detected (Supplementary Fig. 6 J–Q).

To investigate if any epithelial mutations were present in the polyps, next-generation sequencing for common CRC hotspot genes including *Apc*, *Smad4*, *B-raf*, *K-ras*, *N-ras*, *Pik3ca*, *Pten* and *Trp53* was performed. No somatic mutations were detected in 16 polyps that were analyzed. Taken together, these data indicate that the loss of BMPR1A in fibroblasts results in increased MAPK-ERK signaling and is associated with the serrated pathway of CRC development. The absence of a somatic suggests that increased ERK activation is probably induced by soluble factors secreted by the BMPR1A^Δ^ fibroblasts.

### CXCL12 is expressed by intestinal fibroblasts and upregulated in mice with fibroblast-specific BMPR1A knockout

After observing the increased epithelial cell growth, we assumed that this is probably caused by soluble factors specifically derived from BMPR1A KO fibroblasts. We, therefore, combined two publicly available human mRNA-sequencing data sets to identify factors secreted by the tumor stroma (GSE39395), which are also upregulated in serrated polyps (GSE45270) (Supplementary Fig. 7). 99 genes were found to be upregulated in both data sets. The exclusion of genes encoding for intracellular proteins, membrane proteins, and extracellular matrix proteins resulted in a selection of genes encoding for secreted factors (Supplementary Fig. 7B). Subsequent literature research resulted in a shortlist of 10 possible target genes that could influence epithelial cell growth. *CXCL12* and *EFEMP1* belonged to the top 3 most differentially expressed genes in both datasets. qPCR analyses on fibroblasts in which BMP signaling was inhibited, revealed upregulation of CXCL12 to be the strongest (Supplementary Fig. 7C, D), which was therefore selected to evaluate further.

**Fig. 4:**
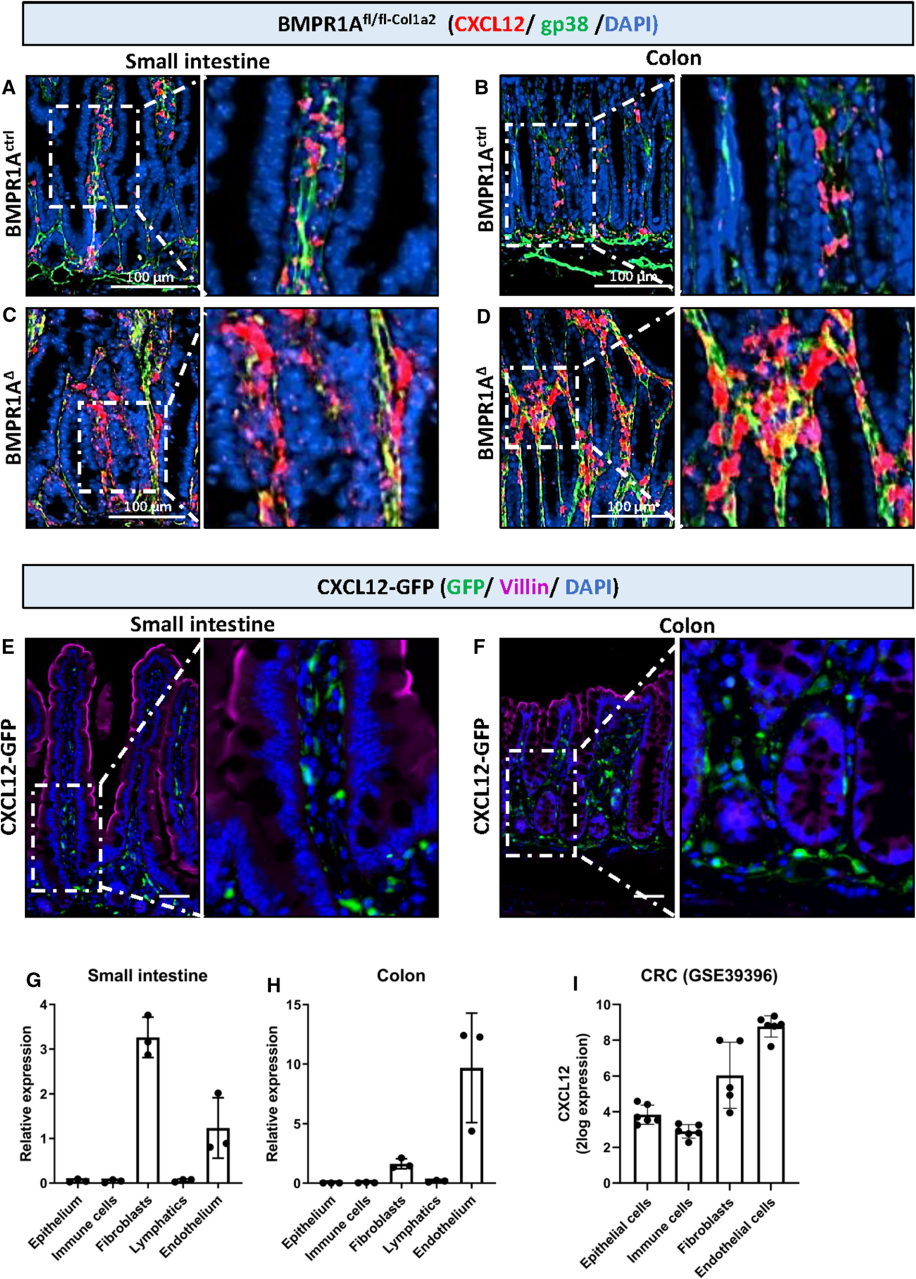
Fibroblast CXCL12 is upregulated in BMPR1A^∆−Col1a2^. Combining two online available mRNA-expression data sets (GSE45270 and GSE39395) identified *CXCL12* as a soluble factor upregulated in CRC tumor stroma and serrated polyps. **A**–**D** RNAscope in situ hybridization for *Cxcl12* and staining for the fibroblast marker gp38 showed an apparent increase of *Cxcl12*-expressing fibroblasts in BMPR1A^∆−Col1a2^ intestinal tissue compared to tissue from BMPR1A^ctrl−Col1a2^. **E**, **F** Immunofluorescent visualization of GFP expressed in intestinal tissue from CXCL12-GFP mice showed that it was only present in the stroma of the small intestine and colon. G-H) qPCR for *Cxcl12* on cell sorted epithelial cells, immune cells, fibroblasts, lymphatic endothelial cells, and endothelial cells isolated from wild-type B6 mice showed that *Cxcl12* is exclusively expressed by fibroblasts and endothelial cells. **I** Human CRC expression data from GSE39396 showed that *CXCL12* is mainly expressed by fibroblasts and endothelial cells and follows the same expression pattern as observed for mouse colonic tissue. Bars represent mean ± SD. *P* < 0.05 (*)

To investigate if CXCL12 could indeed be implicated in epithelial cell growth, RNAscope in situ hybridization for *Cxcl12* was performed on BMPR1A^Δ−Col1a2^ mouse tissue. Increased *Cxcl12* staining was present in the small intestines, polyps, and colon of BMPR1A^∆−Col1a2^ mice compared to control mice (Fig. 4A–D). The membrane marker glycoprotein 38 (gp38) was used to visualize intestinal fibroblasts and showed that *Cxcl12* colocalized in fibroblast-rich regions [19]. To further confirm that intestinal fibroblasts express CXCL12, we used tissue from *Cxcl12*-green fluorescent protein (GFP) transgenic mice in which cells that express CXCL12 also express GFP. Immunofluorescent visualization of GFP showed that CXCL12 is exclusively expressed by the stroma of both the small intestine and the colon (Fig. 4E, F). Immunofluorescent staining for cell-specific markers showed clear co-localization of GFP with endothelial cells (CD31) and general fibroblast markers Collagen-I and gp38, but not with immune cells (CD45) or lymphatic endothelial cells (Lyve1) (Supplementary Fig. 8A). These observations were further validated by performing Fluorescence-Activated Cell Sorting (FACS) on both the small intestine and colon for epithelial cells (EpCam^+^), immune cells (CD45^+^), endothelial cells (gp38^−^ CD31^+^), fibroblasts (gp38^+^CD31^−^), and lymphatic cells (gp38^+^CD31^+^). The gating strategy is indicated in Supplementary Fig. 8B. Real-time quantitative PCR (RT-qPCR) analysis of sorted cells for *Cxcl12* mRNA showed that intestinal *Cxcl12* is only expressed by fibroblasts and endothelial cells. In the small intestines, the highest *Cxcl12* expression was found in fibroblasts, followed by endothelial cells (Fig. 4G). The opposite pattern was found in the colon (Fig. 4H). Interestingly, in colorectal cancer (CRC), *CXCL12* is also expressed most abundantly in endothelial cells, followed by fibroblasts (Fig. 4I). These data show that fibroblasts produce CXCL12 and that expression is increased after the loss of BMPR1A signaling, suggesting that CXCL12 might be involved in the observed phenotype in the BMPR1A^Δ−Col1a2^ mice.

### BMP signaling regulates *CXCL12* expression

**Fig. 5:**
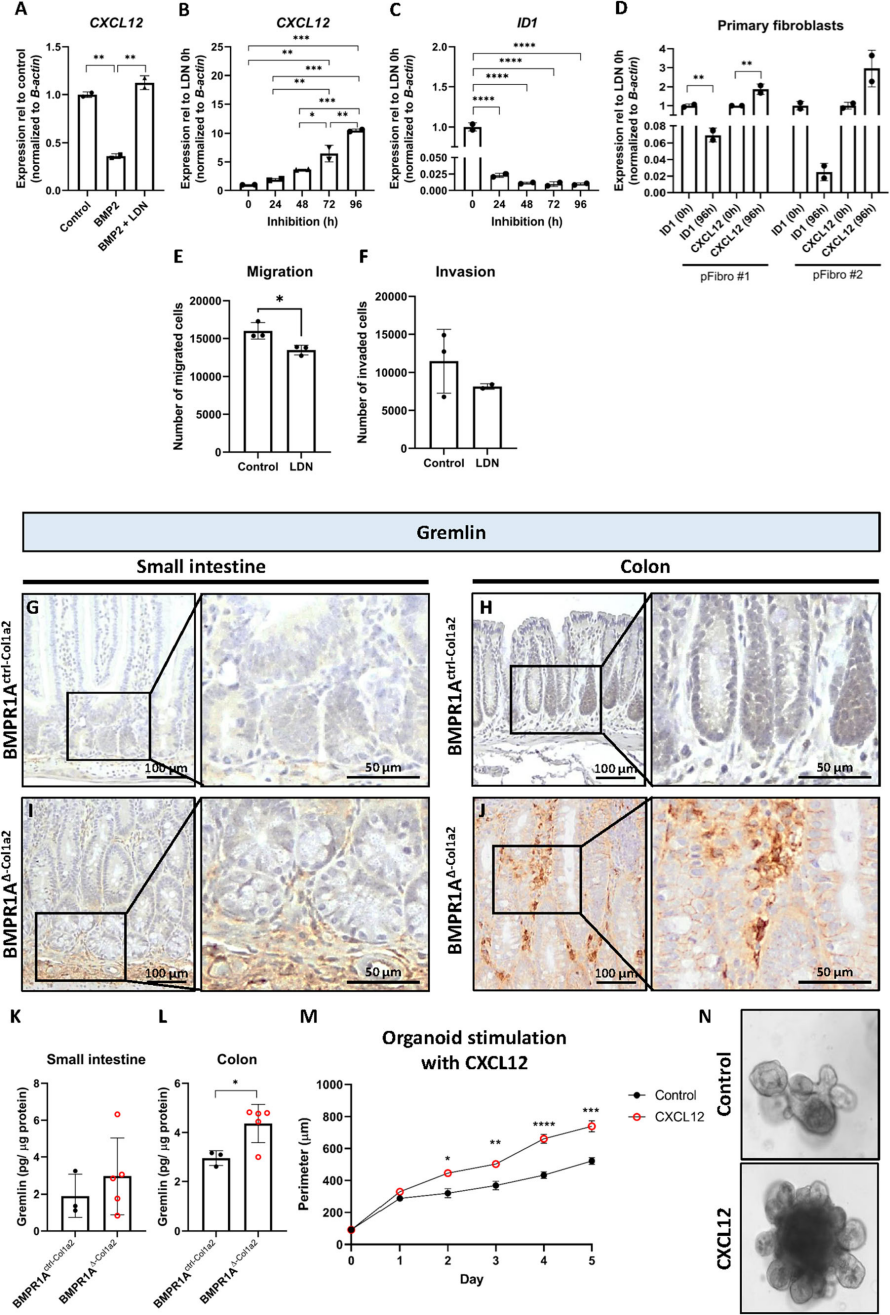
CXCL12 expression is regulated by BMP activity and stimulates epithelial proliferation. **A** Stimulation of CCD-18co fibroblasts with 100 ng BMP2 for 24 h resulted in a significant downregulation of *CXCL12* expression. Downregulation was prevented when 200 nM LDN-193189, a BMPR1A inhibitor, was present in the medium. **B** Stimulating CCD-18co fibroblasts or primary colonic fibroblasts for up to 96 h with 200 nM LDN-193189 showed a gradual increase of *CXCL12* expression over time. **C** Expression of *ID1*, a BMP target gene, decreased, showing that BMP signaling was successfully inhibited. **D** Similar findings were observed when human primary fibroblasts were treated with LDN-193189. **E**, **F** The presence of 200 nM LDN-193189 was found to lower the invasive capacity of fibroblast while also significantly hindering the migratory capacity of the fibroblasts. **G**–**J** Immunohistochemical staining of BMPR1A^∆−Col1a2^ intestinal tissue for Gremlin showed a clear increase compared to the intestines from control mice. **K**–**L** An ELISA for Gremlin confirmed the increased levels in intestines from BMPR1A^∆−Col1a2^ mice. **M**, **N** Treatment of normal intestinal organoids with recombinant CXCL12 showed that it CXCL12 stimulates organoid growth significantly. Bars represent mean ± SD except for (**I**) in which error bars represent mean ± SEM. *P* < 0.05 (*), < 0.01 (**), < 0.001 (***) and < 0.0001 (****)

To investigate how the BMP pathway affects fibroblast *CXCL12* expression, several experiments were performed using the human colon fibroblast CCD-18co. Firstly, stimulation of 18co fibroblasts for 24 h with recombinant BMP2 resulted in a significant decrease of *CXCL12* expression (Fig. 5A, *P* = 0.002). This could be restored by adding LDN-193189, a selective BMPR1A inhibitor. To mimic the long-term effect of loss of BMP signaling, cells were exposed to LDN-193189 for up to 96 h. *CXCL12* expression gradually increased over time, reaching a 10.5-fold increase after 96 h (Fig. 5B). Expression of the BMP target gene *ID1* was downregulated after 24 h and remained downregulated up to 96 h (Fig. 5C), showing that BMP signaling was successfully inhibited over the time course of the experiment. Stimulation of human primary colonic fibroblasts (pFibro) isolated from normal colon tissue for 96 h with LDN-193189 also resulted in increased *CXCL12* expression (Fig. 5D), confirming the findings with the CCD-18co fibroblasts. Besides *CXCL12*, inhibition of BMP signaling was also found to significantly alter expression of *WNT2A, TGF-β2* and *TGF-β3,* but not *TGF-β1* and *A-SMA,*(Supplementary Fig. 6H–K and S).

To further evaluate how inhibition of BMP signaling would influence the functional behavior of fibroblasts, transwell invasion assays were performed. These data showed that inhibition of BMP signaling in fibroblasts with LDN-193189 resulted in decreased migratory and invasive capacities of the fibroblasts (Fig. 5E, F).

BMP antagonists are known to regulate the BMP signaling amplitude in vivo. To investigate whether BMP antagonists can also influence *CXCL12* expression, CCD-18co cells were stimulated for 96 h with the BMP antagonist Noggin. This resulted in *ID1* downregulation and concomitant *CXCL12* upregulation (Supplementary Fig. 9). To explore if the overexpression of BMP antagonists could explain increased *Cxcl12* expression in BMPR1A^∆−Col1a2^ mice in vivo, immunohistochemical stainings were performed for the BMP antagonist Gremlin. Gremlin binds the same BMP ligands, but unlike Noggin, it is almost exclusively expressed by fibroblasts (Supplementary Fig. 10A, B). A substantial increase in Gremlin expression (Fig. 5G–J) in the small and large intestines of the BMPR1A^Δ−Col1a2^ was observed. This was also confirmed in polyps of the BMPR1A^∆−SM22^ mice (Supplementary Fig. 11). ELISA analysis for Gremlin confirmed the increased Gremlin expression in BMPR1A^Δ−Col1a2^ mice (Fig. 5K, L). These data show that the loss of BMPR1A signaling and increased BMP antagonist expression might be instrumental for increased *CXCL12* expression.

To investigate whether CXCL12 indeed stimulates cell growth, we stimulated intestinal mouse organoids with recombinant CXCL12. The organoids were significantly larger from day 2 onwards, compared to unstimulated organoids (Fig. 5M, N). We examined if treatment of organoids with CXCL12 regulated expression of serrated-associated genes *Anxa1*, *Pdx1* (shown to be upregulated in serrated polyps) and *Cdx1* (downregulated in serrated polyps). Treatment of organoids with CXCL12 did not result in significant changes in expression of *Anxa1*, *Cdx1* and *Pdx1* (Supplementary Fig. 12A). Together these data show that loss of BMP signaling can increase *CXCL12* expression, and in turn, CXCL12 can drive epithelial cell growth.

### Treatment of mice with the BMP activator FK-506 or CXCL12 neutraligand LIT-927 abrogates polyp development

**Fig. 6:**
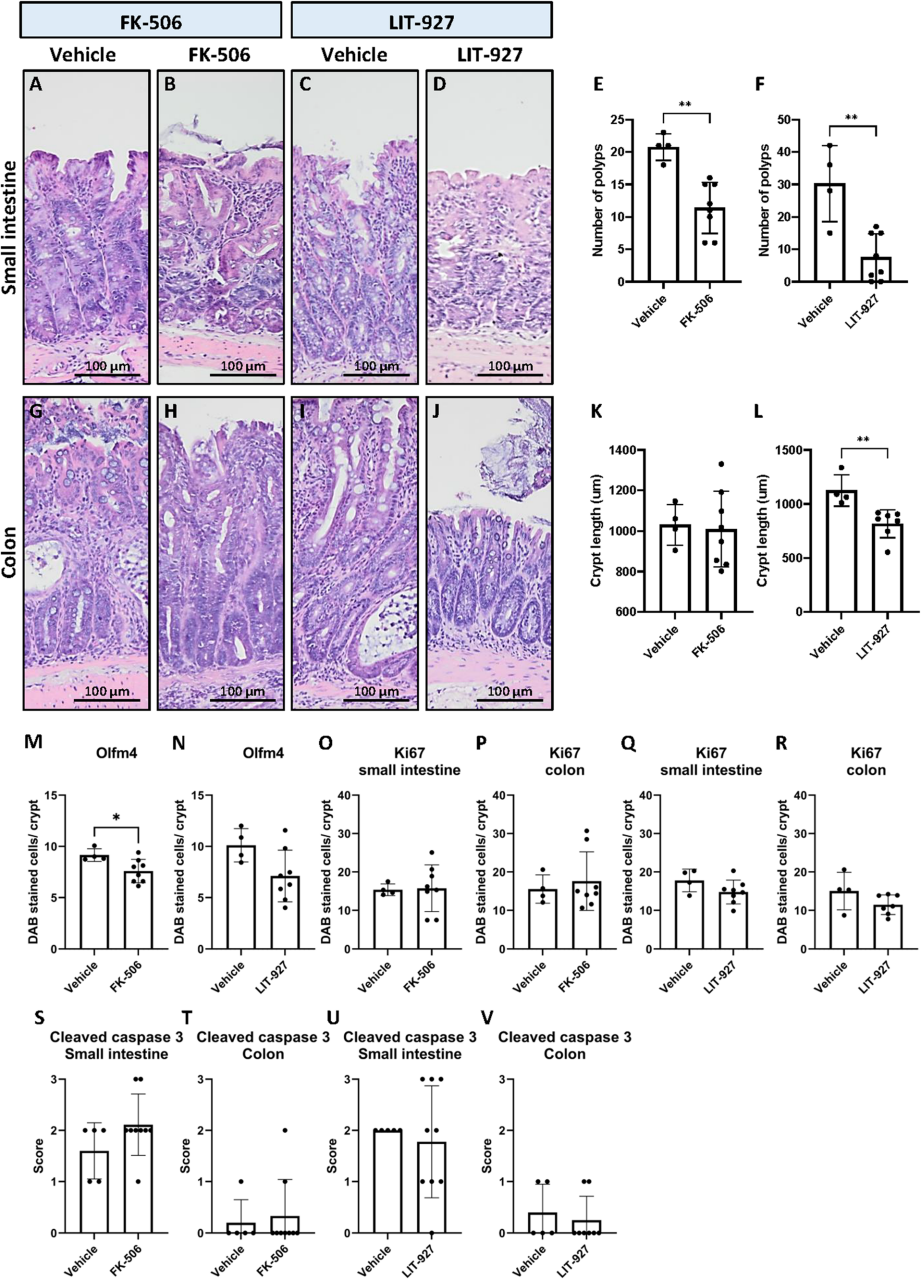
Treatment of mice with the BMP activator FK-506 or the CXCL12 neutralizer LIT-927 results in fewer polyps. **A**–**D** Treatment of mice with FK-506 or LIT-927 resulted in less polyps. **E** 1.8-fold fewer polyps were observed in FK-506 treated mice (mean = 11.4) compared to vehicle-treated mice (mean = 20.8). **F** fourfold fewer polyps were observed in LIT-927 treated mice (mean = 7.5) compared to vehicle control mice (mean = 30.3). **G**–**L** While the FK-506 treatment did not restore the crypt length, a significant decrease in crypt length was observed in mice treated with LIT-927. **M**, **N** Treatment of mice with the compounds resulted in a decreased number of olfm4 positive cells per crypt. This was however only significant in mice treated with FK-506. **O**–**R** The number of Ki67 positive cells showed a decreased trend in mice treated with LIT-927 (not significant). **S**–**V** Treatment of mice with one of the compound did not result in an increase of cells that have undergone apoptosis as assessed with an IHC for cleaved caspase 3. Bars represent mean ± SD. *P* < 0.05 (*) and < 0.01 (**)

Next, we investigated if polyp development in the BMPR1A^Δ−Col1a2^ mice could be abrogated by restoring BMP activity with FK-506, a ligand-independent activator of the BMP pathway. Restoring BMP signaling significantly reduced polyp formation by 45% from 20.8 polyps per mouse in the vehicle control group to an average of 11.4 per mouse in the FK-506 treated group (P = 0.0013, Fig. 6A, B and E). To confirm the role of CXCL12 in polyp development, BMPR1A^Δ−Col1a2^ mice were treated with LIT-927, a CXCL12 neutralizing ligand. A substantial 75% reduction in the number of polyps from 30.3 per mouse to 7.5 per mouse was found for mice treated with LIT-927 compared to mice treated with vehicle (*P* = 0.0018, Fig. 6C, D and F). Next to the decreased number of polyps in the small intestine, LIT-927 normalized the increased the crypt length in the large intestine of treated mice (mean = 817.9 µm, *P* = 0.0054, Fig. 6L) compared to vehicle-treated mice (mean = 1125 µm), while FK-506 was not capable of restoring the increased crypt length (Fig. 6K). These data suggest that inducing BMP activity with FK-506 or inhibiting CXCL12 signaling due to neutralization of CXCL12 can significantly prevent polyp development in BMPR1A^Δ−Col1a2^ mice.

Next, we investigated the effect of the treatments on the number of Olfm4, Ki67, CD45 and cleaved caspase 3 positive cells. The number of Ki67 positive cells and percentage of CD45 positive cells seemed to decrease in the treated mice, although this was not significant (Fig. 6O–R and Supplementary Fig. 13 and 14). In contrast, significantly less Olfm4 positive cells per crypt were observed in mice treated with FK-506 (Fig. 6M). A similar decrease was observed for mice treated with LIT-927 but due to the increased variation between mice this was found to be not significant (Fig. 6N). No significant changes in the number of apoptotic cells were observed based on the cleaved caspase 3 staining (Fig. 6S–V).

### CMS4 colorectal cancers express high levels of BMP antagonists and CXCL12

**Fig. 7:**
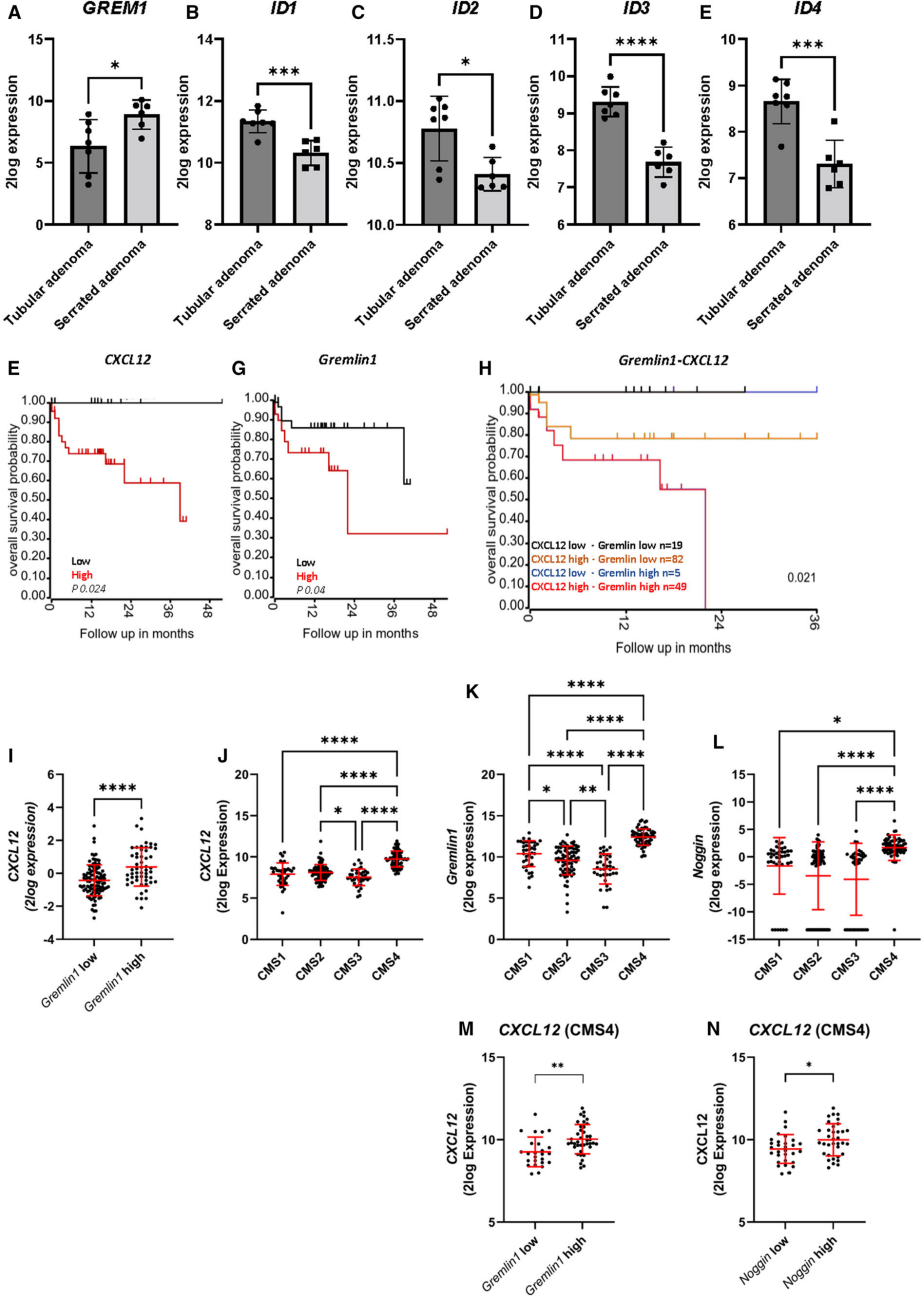
*CXCL12* and the BMP antagonists *GREMLIN1* and *NOGGIN* are significantly upregulated in CMS4 tumors. **A**–**E** Serrated adenomas express *GREMLIN1* compared to tubular adenomas. Several ID proteins were found to be lower expressed in serrated adenomas. **F**–**G** Analyzing a colon adenocarcinoma TCGA cohort (*n* = 155) for *GREMLIN1* and *CXCL12* showed that patients with high *GREMLIN1* or *CXCL12* had a significantly poorer survival compared to patients with low expression. **H** Stratifying the 155 samples for both *CXCL12* and *GREMLIN1*, showed that particularly patients with high expression of both *CXCL12* and *GREMLIN1* had a significantly worse overall survival. **I** High *GREMLIN1* expression showed a significant association with higher *CXCL12* expression **J**–**L** Stratification of colorectal TCGA cases (*n* = 240) into the four CMS subtypes showed that *GREMLIN1*, *NOGGIN* and *CXCL12* are significantly upregulated in CMS4 tumors. **M**, **N** High *GREMLIN1* and *NOGGIN* were significantly associated with higher *CXCL12* levels. Bars represent mean ± SD. *P* < 0.05 (*), < 0.01 (**), < 0.001 (***) and < 0.0001 (****)

To investigate if decreased BMP signaling is also implicated in the development of human serrated polyps, publicly available human mRNA-sequencing data sets were used in which the expression profile of serrated polyps are compared to tubular adenomas (GSE45270). *GREMLIN1* expression was significantly upregulated, while the inhibitor of DNA binding (ID) proteins ID1, ID2, ID3 and ID4, all major downstream transcription targets of BMP signaling, were found to be significantly downregulated in serrated adenomas compared to tubular adenomas. This suggests that BMP activity is indeed decreased in serrated adenomas (Fig. 7A–E).

Next, to study the relation between BMP antagonist expression and CXCL12 in humans, a publicly available the cancer genome atlas (TCGA) mRNA-expression data set from a colon adenocarcinoma cohort with survival data consisting of 155 patients was used. High *GREMLIN1* and high *CXCL12* expression were associated with a significantly poorer overall survival (*P* = 0.04 and P = 0.024 respectively, Fig. 7F–G). While a similar pattern was observed for *NOGGIN* in the first 24 months of follow-up, this did not reach statistical significance (*P* = 0.116, Supplementary Fig. 15A). High *GREMLIN1* levels were associated significantly (*P* ≤ 0.0001) with higher *CXCL12* expression while *NOGGIN* was not (Fig. 7I and Supplementary Fig. 15B). Moreover, patients with tumors expressing high levels of both *GREMLIN1* and *CXCL12* had a significantly worse survival (*P* = 0.021) compared to patients with high expression of only *CXCL12*, *GREMLIN1,* or *NOGGIN* (Fig. 7H). Similar trends were observed for *NOGGIN,* but these did not reach statistical significance (*P* = 0.095, Supplementary Fig. 15C).

Since the BMP antagonists are expressed by stromal cells in the colon, we hypothesized that the BMP-CXCL12 signaling axis would be of particular significance in tumors with an abundant stromal compartment. The Consensus Molecular Subtype (CMS) classification system subdivides CRCs into four subgroups based on the transcriptional signature to which both cancer cells and the tumor stroma contribute [16]. The CMS4 tumors are known as the mesenchymal subtype because of gene signatures consistent with activated tumor stroma, such as active TGF-β signaling. We used TCGA RNA sequence data from sporadic CRC patients to look at the expression of *GREMLIN1*, *NOGGIN,* and *CXCL12* between the 4 different CMS subgroups. Both the BMP antagonists and *CXCL12* were more highly expressed in CMS4 compared to CMS1-CMS3 (Fig. 7J–L). Dividing patients into *GREMLIN1* low and *GREMLIN1* high expressing subgroups or *NOGGIN* low and *NOGGIN* high expressing subgroups showed that patients with high *GREMLIN1* or *NOGGIN* also had significantly higher *CXCL12* expression (Fig. 7M, N). These data together support the existence of a tumor-promoting BMP-CXCL12 signaling axis.

## Discussion

This study shows that loss of BMPR1A, specifically in fibroblasts or myofibroblasts but not endothelial cells, results in CXCL12-driven epithelial hyperproliferation and serrated polyp formation, emphasizing the role of stromal BMP signaling in intestinal homeostasis and serrated polyp development.

Stromal cells have been shown to be important in maintaining intestinal homeostasis by regulating epithelial cell proliferation and differentiation. Loss of BMPR1A in mesenchymal cells results in reactive stroma and subsequent polyposis, supporting the idea that BMP signaling loss in the stroma is sufficient to initiate polyp development [20]. While these studies provide clues that the stroma is involved, it remains unclear which cells are crucial as these studies do not pinpoint the changes to a specific stromal cell type [20, 21]. This study shows that BMP signaling specifically in *Col1a2* fibroblasts or *SM22* expressing myofibroblasts, but not endothelial cells, is a crucial regulator of intestinal homeostasis. Loss of BMPR1A in *Col1a2* expressing cells resulted in major histological changes in the colon and serrated polyp development in the small intestine.

The underlying molecular mechanism behind the polyp development that occurs upon loss of BMP signaling is unknown. Here we report a notable increase of *Cxcl12*-expressing cells in the intestines from BMPR1A^∆−Col1a2^ mice and show that inhibition of BMP signaling, either by the loss of the receptor or antagonist overexpression, resulted in *CXCL12* upregulation in vitro. CXCL12 is a homeostatic chemokine, identified initially in regulating hematopoietic cell trafficking and the organization of secondary lymphoid tissue [22]. This might explain the significant influx of CD45 positive cells in the colon of the BMPR1A^∆−Col1a2^ mice (Supplementary Fig. 4), next to the effects on the epithelial cells as shown in this study. One of the receptors for CXCL12, CXCR4, can be found on epithelial cells of many different organs, such as that of the intestines [23]. Interestingly, binding of CXCL12 to its receptors is known to activate the MAPK/ERK signaling cascade, possibly explaining the increased pERK staining in the intestinal epithelial cells of BMPR1A^∆−Col1a2^ mice [24]. For most cancers, including CRC, CXCR4-CXCL12 signaling has mostly been investigated in the context of tumor progression and metastasis, but not in the early stages of carcinogenesis [25, 26]. Our data show that CXCL12 can stimulate the proliferation of non-cancerous epithelial organoids, indicating that fibroblast-derived CXCL12 could drive epithelial proliferation and potentially polyp initiation upon loss of BMPR1A signaling in fibroblasts. Importantly, we show that neutralizing CXCL12 with LIT-927 significantly reduces polyp development in BMPR1A^∆−Col1a2^ mice, suggesting that CXCL12 is indeed involved in polyp initiation. In addition, re-activating BMP signaling with FK-506 also resulted in significantly fewer polyps and suggests that polyp development can also be prevented by restoring BMP signaling. From the two drugs, FK-506 and LIT-927, LIT-927 seemed to be more effective. In addition to preventing polyp development more efficiently, LIT-927 was also able to partially restore the crypt length. This might be due to the different mechanism of action of the two compounds. LIT-927 is a neutralizing ligand that targets CXCL12 specifically. FK-506, on the other hand, is a BMP analog that works through FKBP12 antagonism that is downstream of the BMP receptors.

Recent data have proposed that serrated polyps are likely to be the precursor lesions from which CMS4 CRCs arise [16]. Interestingly, we found significantly higher *GREMLIN1*, *NOGGIN,* and *CXCL12* levels in mesenchymal or CMS4 CRCs. As has been firmly established, BMP activity is strongly influenced by its antagonists [4, 27]. The increased expression of the BMP antagonists could contribute in different ways to the increased proliferation of epithelial cells, both via induction of *CXCL12* upregulation but also directly cells by inhibiting the suppression of the BMP-mediated restriction of Lgr5 + stem cell signature genes [28]. Probably both can contribute to serrated polyp development.

A previous publication reported that the poor-prognostic CMS4 subtype is characterized by high CXCL12 levels expressed specifically by fibroblasts [29]. However, the mechanism behind CXCL12 upregulation remained unclear but might again be explained by BMP- CXCL12 signaling axis. To our knowledge, there are no papers linking decreased BMP activity to increased CXCL12 expression in cancer. These results suggest that the BMP- CXCL12 signaling axis might be implicated in serrated polyp development. In addition, we found that high *GREMLIN1* and *NOGGIN* expression levels were associated with higher *CXCL12* expression and that patients with both high BMP antagonist levels and *CXCL12* expression levels had a poor overall survival. However, it remains to be elucidated if the BMP-CXCL12 signaling axis is also of significance in JPS. The polyps formed in individuals with JPS have been characterized as hamartomatous polyps, while the polyps that have developed in the BMPR1A^∆−Col1a2^ mice have a serrated histology. This could suggest that the mechanism behind the polyp formation is distinct. Transcriptional and protein analyses of polyp specimens from JPS patients should indicate if this is indeed the case.

Taken together, our study shows that fibroblasts with impaired BMP signaling upregulate CXCL12 expression, which is involved in serrated polyp development in the mouse intestine. As CXCL12 can facilitate epithelial proliferation, the altered BMP-CXCL12 signaling possibly contributes to the progression of serrated polyps.

## Supplementary Information

Below is the link to the electronic supplementary material.

Supplementary file1 (DOCX 16 KB)

Supplementary file2 (DOCX 14 KB)

Supplementary file3 (DOCX 14 KB)

Supplementary file4 (CSV 22 KB)

Supplementary file5: Supplementary figure 1. Loss of BMPR1A signaling in endothelial cells does not result in histological changes. A-H) No histological changes were observed in the intestines of mice. EYFP staining in endothelial cells showed efficient endothelial specific recombination. (TIF 1898 KB)

Supplementary file6: Supplementary figure 2. Efficient recombination in BMPR1A∆-Col1a2 mice. A-E) Immunohistochemical staining for the EYFP protein showed the presence of EYFP-positive cells scattered throughout the lamina propria of the small intestine, polyps and colon of BMPR1A∆-col1a2 mice but not in BMPR1Actrl-Col1a2 mice. F-J) some EYFP-positive cells are found scattered throughout the lamina propria, but the most EYFP-positive cells were found in the submucosa and smooth muscle cells of the small intestine, polyps, and colon of BMPR1A∆-SM22 mice. (TIF 2252 KB)

Supplementary file7: Supplementary figure 3. Loss of BMPR1A signaling resulted in histological changes. A) The colon crypts were found to be longer compared to control mice. B) A RT-qPCR for Col1a2 showed a ~ 4-fold higher Col1a2 expression in the colon compared to the small intestine. C-G) The number of EYFP-positive cells (% DAB-positive area) was higher in the colon compared to the small intestine. Bars represent mean ± SD. P <0.05 (*), <0.01(**), <0.001(***) and <0.0001(****).(TIF 2328 KB)

Supplementary file8: Supplementary figure 4. Loss of BMPR1A signaling resulted in histological changes in intestines of BMPR1A∆-Col1a2 mice. A-N) In the small intestines, no differences were observed for goblet cell numbers as judged by Alcian Blue staining, and CD45 numbers as judged by CD45 immunohistochemistry whereas significant changes were observed in the colon. I) The percentage of apoptotic cells was scored according to a scoring system based on the percentage of cells positive for cleaved caspase 3. J-K) Cleaved caspase 3 was found to be significantly increased in the colon of BMPR1A∆-col1a2 mice compared to control mice. P <0.05 (*), <0.01(**), <0.001(***) and <0.0001(****). (TIF 2635 KB)

Supplementary file9: Supplementary figure 5. Loss of BMPR1A signaling resulted in histological changes in intestines of BMPR1A∆-SM22 mice. A-D) The loss of BMPR1A signaling resulted in an increase of Ki67+ cells, but no differences were observed for the number of goblet cells (E-H), vimentin (I-L) and CD45 (M-P). Bars represent mean ± SD. P <0.05 (*), <0.01(**), <0.001(***) and <0.0001(****). (TIF 3057 KB)

Supplementary file10: Supplementary figure 6. Loss of BMPR1A signaling resulted in increased TGF-β expression. A-E) TGF-β was found to be increased in the intestine of BMPR1A∆-col1a2 mice compared to control mice. Although also increased in the small intestine, the increase was found to be only significant in the colon. F-I) Stimulation of CCD-18co fibroblasts with 200 nM LDN-193189 for up to 96 hours showed that fibroblasts alter their TGFβ1, TGFβ2 and TGFβ3 expression significantly when BMP signaling is inhibited. The expression of WNT2A increased gradually but significantly over time. J-P) No changes were observed in the percentage of α-SMA positive expression between BMPR1A∆-col1a2 mice and control mice. Q) Stimulation of CCD-18co with LDN-193189 also did not result in any significant changes of A-SMA expression. Bars represent mean ± SD. P <0.05 (*), <0.01(**) and <0.001(***) (TIF 2711 KB)

Supplementary file11: Supplementary figure 7. CXCL12 is a factor specifically upregulated in serrated polyps and tumor stroma. A) The combination of two online publicly available data sets, GSE45270 and GSE39395, identified 384 differently expressed genes in both sets. From these 384 genes, 99 encoded for a secreted protein. B) From these 99 factors, 10 were expected to have an effect on epithelial cells. CXCL12 belonged to the top 3 most differentially expressed genes. C-D) Stimulation of CCD-18co fibroblasts for up to 96 hours with 200 nM LDN-193189 resulted in a significant increase of CXCL12 expression reaching almost 8-fold after 96 hours. EFEMP1 was also found to be significantly upregulated with a 2-fold increase in expression after 96 hours. Bars represent mean ± SD. P <0.05 (*), <0.01(**) and <0.001(***). (TIF 365 KB)

Supplementary file12: Supplementary figure 8. CXCL12 expression is present in fibroblasts and endothelial cells but not immune cells and lymphatic endothelial cells. Immunofluorescent visualization of GFP in intestinal tissue from CXCL12-GFP mice showed that CXCL12-GFP was only present in the stroma of the small intestine and colon. A) CXCL12 expression co-localized with endothelial cells (CD31+) and fibroblasts (gp38+ and Collagen I+) but not with lymphatic endothelial cells (Lyve1+) and leukocytes (CD45+). B) FACS sort strategy for sorting epithelial cells, immune cells, fibroblasts, endothelial cells and lymphatic endothelial cells. (TIF 3840 KB)

Supplementary file13: Supplementary figure 9. BMP antagonists also regulate CXCL12 expression. A) Stimulation of 18co fibroblasts with 100 ng BMP2 for 24h resulted in a significant downregulation of CXCL12. Downregulation was prevented when 200 ng recombinant Noggin was present in the medium. B) Stimulating CCD-18co fibroblasts or primary colonic fibroblasts for up to 96h with Noggin showed a gradual increase of CXCL12 over time. C) ID1 expression also decreased, showing that BMP signaling was successfully prevented throughout the stimulation. Bars represent mean ± SD. P <0.05 (*), <0.01(**), <0.001(***) and <0.0001(****). (TIF 319 KB)

Supplementary file14: Supplementary figure 10. Expression of GREMLIN1 and NOGGIN by different cell types in CRC. A) GREMLIN1 gene expression was higher in the fibroblast population compared with leukocytes, endothelial cells, and epithelial cells isolated by FACS (GSE39396). B) NOGGIN gene expression was found to be high in both fibroblasts and leukocytes compared with endothelial cells and epithelial cells. (TIF 338 KB)

Supplementary file15: Supplementary figure 11. Loss of BMPR1A signaling resulted in higher Gremlin expression in the intestines of BMPR1A∆-SM22 mice. A-D) A clear increase of Gremlin expression was observed in KO mice compared to controls as assessed by immunohistochemistry using an anti-GREM1 antibody. (TIF 865 KB)

Supplementary file16: Supplementary figure 12. Stimulation of intestinal organoids with CXCL12 does not lead to significant changes in expression of genes associated with the serrated phenotype. A) stimulation of intestinal organoids with CXCL12 did results in changes of expression of Pdx1, Anxa1 and Cdx1. (TIF 320 KB)

Supplementary file17: Supplementary figure 13. Treatment of BMPR1A∆-Col1a2 mice with FK-506 leads to significant fewer olfm4 positive cells per crypt. A-L) Representative images of olfm4 and ki67 stainings taken from the mice treated with FK-506, LIT-927 or vehicle controls. (TIF 4510 KB)

Supplementary file18: Supplementary figure 14. BMPR1A∆-Col1a2 mice with FK-506 or LIT-927 does not lead to significant changes in the percentage of CD45 cells. A-L) While treatment of mice with FK-506 or LIT-927 resulted in a decrease of CD45 cells in the intestinal tissue, this decrease was found to be not significant. (TIF 3606 KB)

Supplementary file19: Supplementary figure 15. NOGGIN expression is not significantly associated with patient survival. A) Stratification of NOGGIN in low and high expressing groups does not show a significant difference in patient survival between the groups. B) No association was found between NOGGIN expression and CXCL12 expression. C) Stratification of patients for NOGGIN and CXCL12 expression does not show a significant difference in patient survival between the groups. (TIF 326 KB)
