# Supplementary material for: Loss of bone morphogenetic protein signaling in fibroblasts results in CXCL12-driven serrated polyp development
Source: J Gastroenterol. 2022 Nov 3;58(1):25–43. doi: 10.1007/s00535-022-01928-x (PMC9825358; doi:10.1007/s00535-022-01928-x)
Supplement: Supplementary file 1 — Supplementary file1 (DOCX 16 KB) [file 535_2022_1928_MOESM1_ESM.docx]

Supplementary Table 1 Dilution of primary antibodies.

| Marker | Target | Use | Primary Ab dilution | Clone or catalog# | Company |
| --- | --- | --- | --- | --- | --- |
| B-catenin | B-catenin | IHC | 1/100 | Clone 14/Beta-Catenin | BD Biosciences |
| CD45 | Immune cells | IHC | 1/3200 | 30-F11 | eBioscience |
| Cleaved caspase 3 | Apoptotic cells | IHC | 1/1200 | ab32350 | Cell signaling Technology |
| GFP | EYFP | IHC | 1/2000 | 600-101-215 | Rockland |
| Gremlin1 | Gremlin1 | IHC | 1/200 | BAF956 | R&D systems |
| Goat- α- rabbit | Primary Ab | IHC | 1/200 | E0432 | Dako |
| Goat- α-mouse | Primary Ab | IHC | 1/200 | E0433 | Dako |
| Rabbit- α-goat | Primary Ab | IHC | 1/200 | E0466 | Dako |
| Ki67 | Proliferating cells | IHC | 1/300 | SP6 | Abcam |
| Olfm4 | Stem cells | IHC | 1/800 | D6Y5A | Cell signaling Technology |
| pERK | pERK1/2 | IHC | 1/3200 | 9101 | Cell signaling Technology |
| Vimentin | Stromal cells | IHC | 1/400 | D21H3 | Cell signaling Technology |
| Collagen I | Fibroblasts | IF | 1/200 | 1330-01 | Southern Biotech |
| GFP | GFP | IF | 1/75 | D5.1 | Cell signaling Technology |
| gp38 | Fibroblasts | IF | 1/50 | AF3244 | R&D systems |
| Villin | Epithelial cells | IF | 1/50 | C-19 | Santa Cruz |
| CD31 | Endothelial cells | FACS | 1/400 | 390 | eBioscience |
| CD45 | Immune cells | FACS | 1/800 | 30-F11 | eBioscience |
| EpCam | Epithelial cells | FACS | 1/400 | G8.8 | Santa Cruz |
| gp38 | Fibroblasts | FACS | 1/400 | 8.1.1 | Biolegend |
