# Supplementary material for: Loss of bone morphogenetic protein signaling in fibroblasts results in CXCL12-driven serrated polyp development
Source: J Gastroenterol. 2022 Nov 3;58(1):25–43. doi: 10.1007/s00535-022-01928-x (PMC9825358; doi:10.1007/s00535-022-01928-x)
Supplement: Supplementary file 2 — Supplementary file2 (DOCX 14 KB) [file 535_2022_1928_MOESM2_ESM.docx]

Table 2 Components of the ENR organoid culture medium

| Factor | Firm | Final concentration |
| --- | --- | --- |
| Advanced DMEM/F12 | Gibco | - |
| HEPES 100x | Gibco | 1x |
| GlutaMax 100x | Gibco | 1x |
| Penicillin-streptomycin | Gibco | 1x |
| B27 supplement 50x | Gibco | 1x |
| recombinant Noggin | Peprotech | 100 ng/ml |
| recombinant R-spondin | Peprotech | 100 ng/ml |
| Wnt-surrogate | U-Protein Express BV | 120 ng/ml |
| N-Acetyl-L-cysteine | Merck | 1.25 mM |
| Nicotinamide | Merck | 10 mM |
| mEGF | Gibco | 5 ng/ml |
| A83-01 | Merck | 500 nM |
| SB202190 | Gentaur | 10 µM |
