# Supplementary material for: Loss of bone morphogenetic protein signaling in fibroblasts results in CXCL12-driven serrated polyp development
Source: J Gastroenterol. 2022 Nov 3;58(1):25–43. doi: 10.1007/s00535-022-01928-x (PMC9825358; doi:10.1007/s00535-022-01928-x)
Supplement: Supplementary file 3 — Supplementary file3 (DOCX 14 KB) [file 535_2022_1928_MOESM3_ESM.docx]

Table 3 Primer sequences

| Gene | Forward primer | Reverse primer |
| --- | --- | --- |
| *Anxa1* mouse | TGTATCCTCGGATGTTGCTGCC | CCATTCTCCTGTAAGTACGCGG |
| *B-actin* mouse | AGGTCATCACTATTGGCAACGA | CCAAGAAGGAAGGCTGGAAAA |
| *B-ACTIN* human | GCAGGCACTCAGGTCAG | ATTCGGTGATGGAAACTGC |
| *Cdx1* mouse | CAAGGCGGACGCCCTACGAAT | TAGGCGTTGGTGGTCTGTGTAG |
| *Cxcl12* mouse | TTTCAGATGCTTGACGTTGG | GCGCTCTGCATCAGTGAC |
| *CXCL12* Human | CTCCACATCCTCCACGTTCT | GCTTTGGTCCTGAGAGTCCT |
| *Col1a2* mouse | CCCAGAGTGGAACAGCGATT | ATGAGTTCTTCGCTGGGGTG |
| *ID1* human | CAGCCAGTCGCCAAGAAT | ACAGACAGCGCACCACCT |
| *Pdx1* mouse | TTCCCGAATGGAACCGAGCCTG | TTTTCCTCGGGTTCCGCTGTGT |
