# Supplementary figures and images for: Loss of bone morphogenetic protein signaling in fibroblasts results in CXCL12-driven serrated polyp development
Source: J Gastroenterol. 2022 Nov 3;58(1):25–43. doi: 10.1007/s00535-022-01928-x (PMC9825358; doi:10.1007/s00535-022-01928-x)

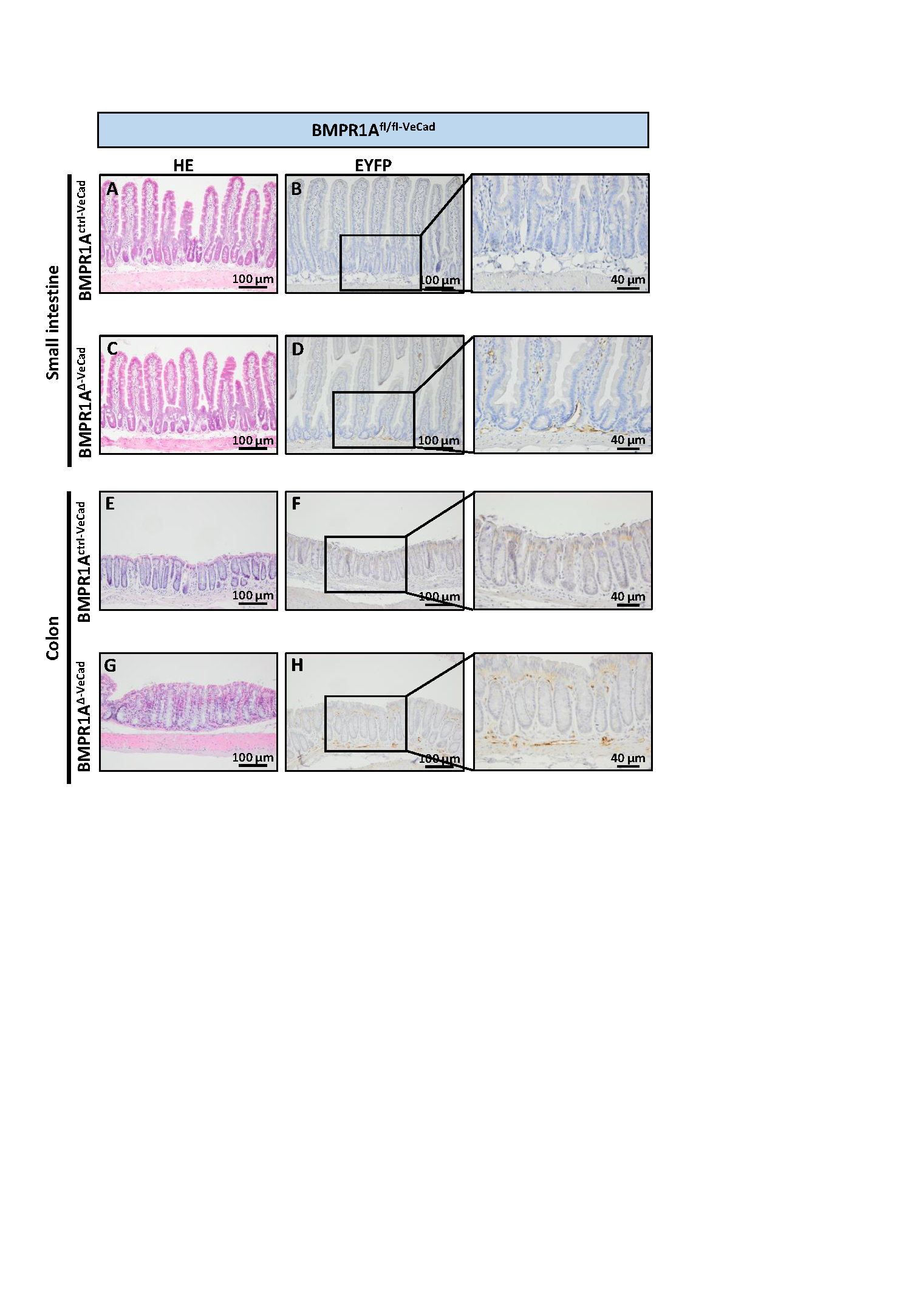

Supplement: Supplementary file 5 — Supplementary file5: Supplementary figure 1. Loss of BMPR1A signaling in endothelial cells does not result in histological changes. A-H) No histological changes were observed in the intestines of mice. EYFP staining in endothelial cells showed efficient endothelial specific recombination. (TIF 1898 KB) [file 535_2022_1928_MOESM5_ESM.tif]

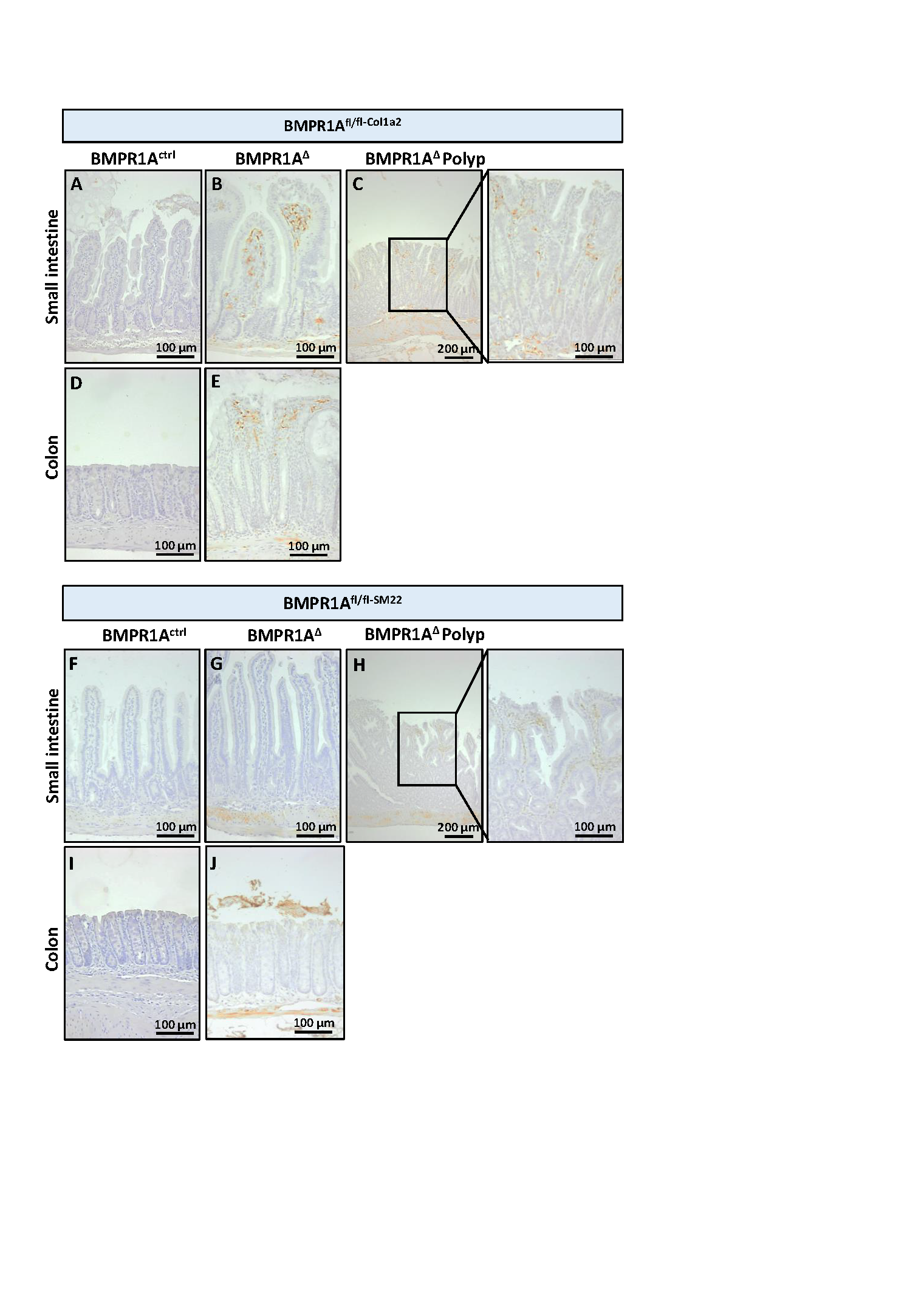

Supplement: Supplementary file 6 — Supplementary file6: Supplementary figure 2. Efficient recombination in BMPR1A∆-Col1a2 mice. A-E) Immunohistochemical staining for the EYFP protein showed the presence of EYFP-positive cells scattered throughout the lamina propria of the small intestine, polyps and colon of BMPR1A∆-col1a2 mice but not in BMPR1Actrl-Col1a2 mice. F-J) some EYFP-positive cells are found scattered throughout the lamina propria, but the most EYFP-positive cells were found in the submucosa and smooth muscle cells of the small intestine, polyps, and colon of BMPR1A∆-SM22 mice. (TIF 2252 KB) [file 535_2022_1928_MOESM6_ESM.tif]

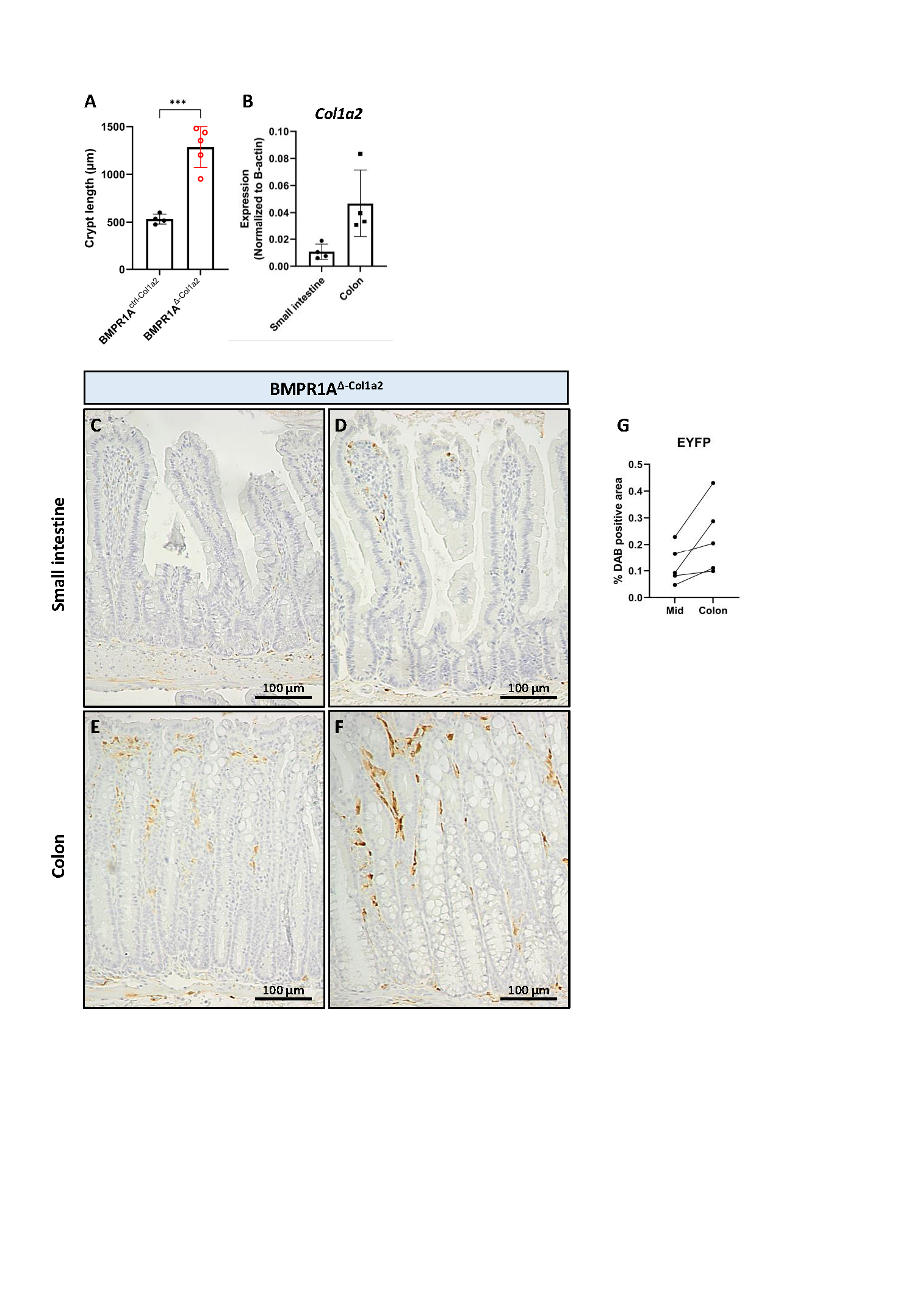

Supplement: Supplementary file 7 — Supplementary file7: Supplementary figure 3. Loss of BMPR1A signaling resulted in histological changes. A) The colon crypts were found to be longer compared to control mice. B) A RT-qPCR for Col1a2 showed a ~ 4-fold higher Col1a2 expression in the colon compared to the small intestine. C-G) The number of EYFP-positive cells (% DAB-positive area) was higher in the colon compared to the small intestine. Bars represent mean ± SD. P <0.05 (*), <0.01(**), <0.001(***) and <0.0001(****).(TIF 2328 KB) [file 535_2022_1928_MOESM7_ESM.tif]

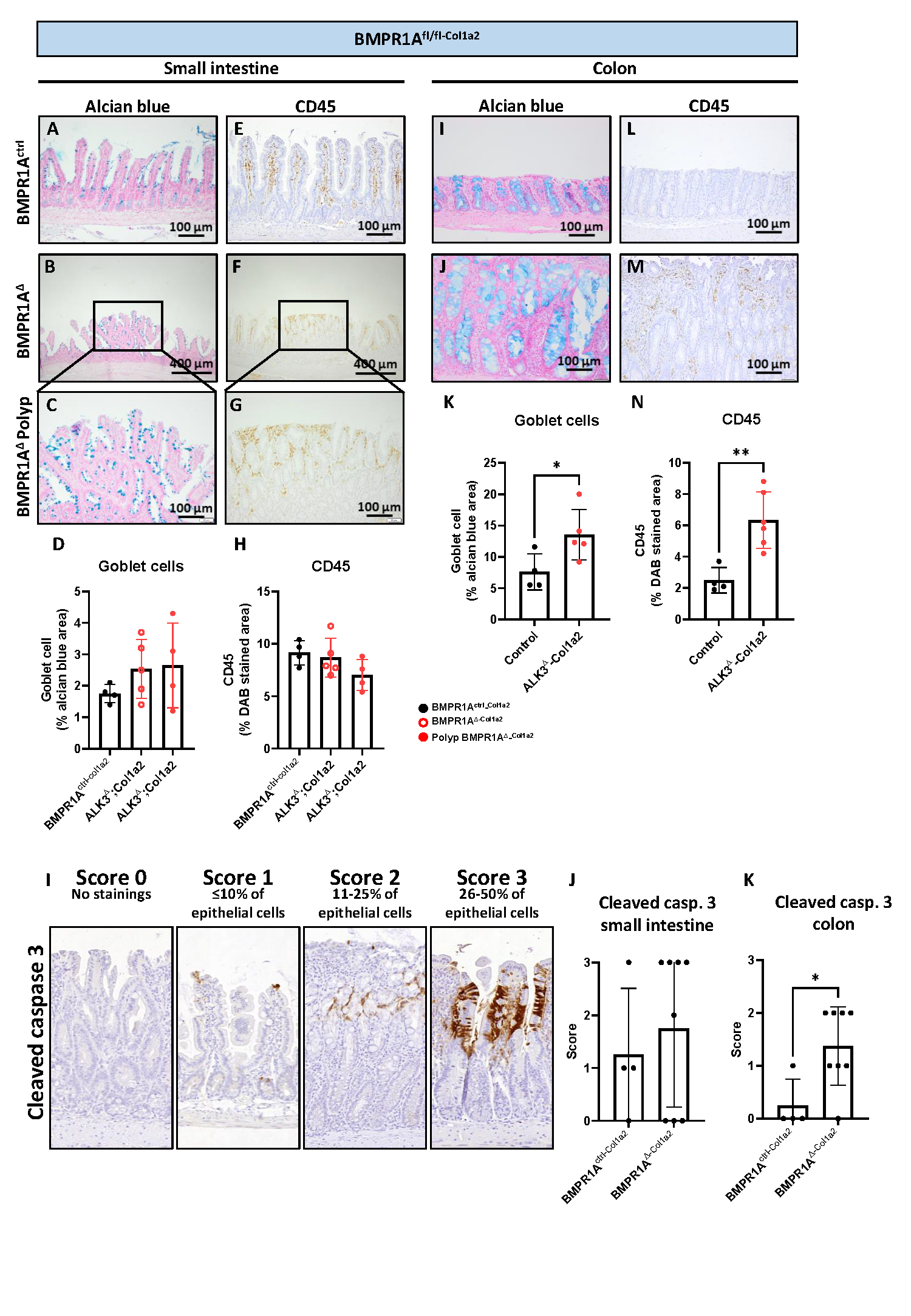

Supplement: Supplementary file 8 — Supplementary file8: Supplementary figure 4. Loss of BMPR1A signaling resulted in histological changes in intestines of BMPR1A∆-Col1a2 mice. A-N) In the small intestines, no differences were observed for goblet cell numbers as judged by Alcian Blue staining, and CD45 numbers as judged by CD45 immunohistochemistry whereas significant changes were observed in the colon. I) The percentage of apoptotic cells was scored according to a scoring system based on the percentage of cells positive for cleaved caspase 3. J-K) Cleaved caspase 3 was found to be significantly increased in the colon of BMPR1A∆-col1a2 mice compared to control mice. P <0.05 (*), <0.01(**), <0.001(***) and <0.0001(****). (TIF 2635 KB) [file 535_2022_1928_MOESM8_ESM.tif]

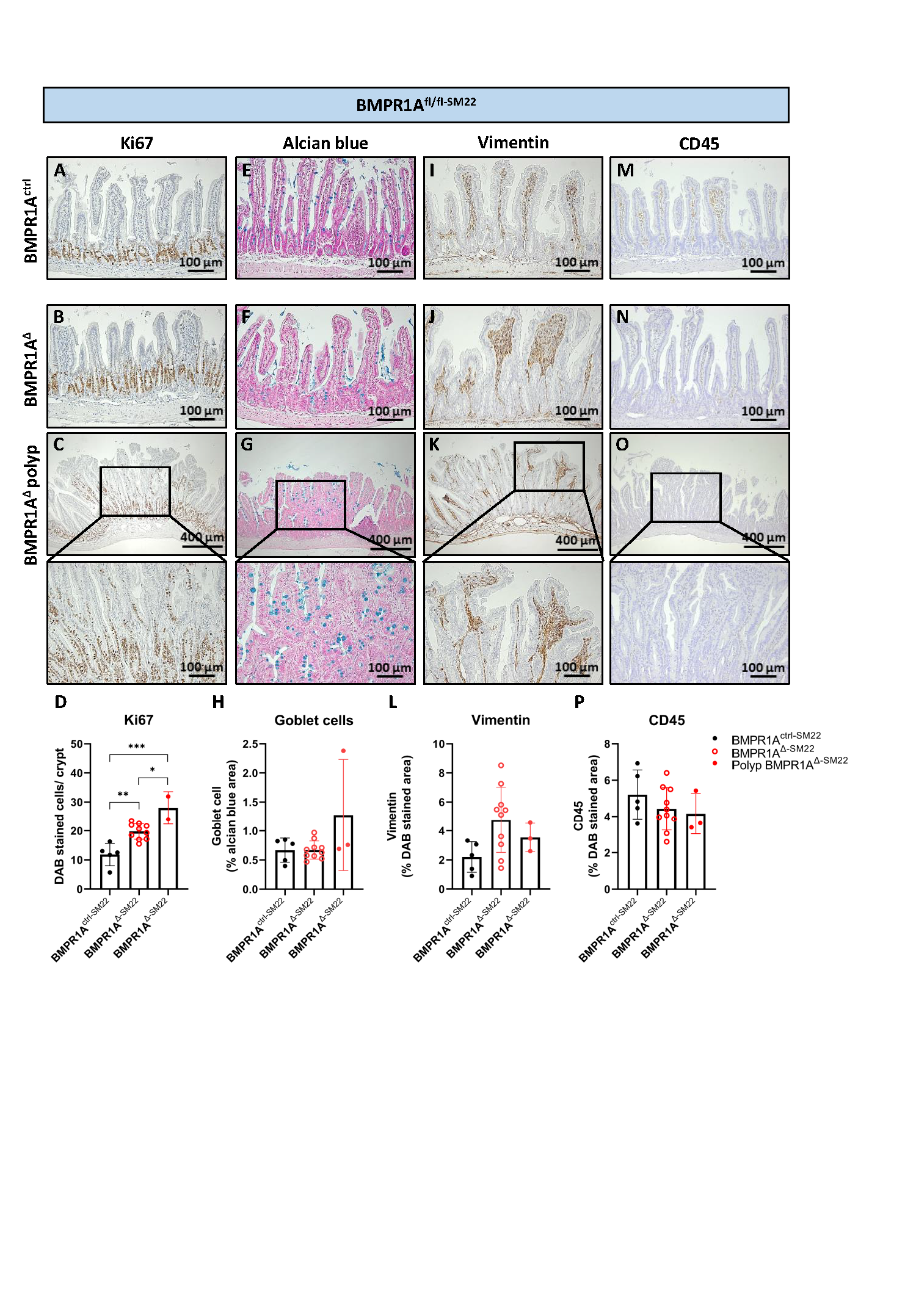

Supplement: Supplementary file 9 — Supplementary file9: Supplementary figure 5. Loss of BMPR1A signaling resulted in histological changes in intestines of BMPR1A∆-SM22 mice. A-D) The loss of BMPR1A signaling resulted in an increase of Ki67+ cells, but no differences were observed for the number of goblet cells (E-H), vimentin (I-L) and CD45 (M-P). Bars represent mean ± SD. P <0.05 (*), <0.01(**), <0.001(***) and <0.0001(****). (TIF 3057 KB) [file 535_2022_1928_MOESM9_ESM.tif]

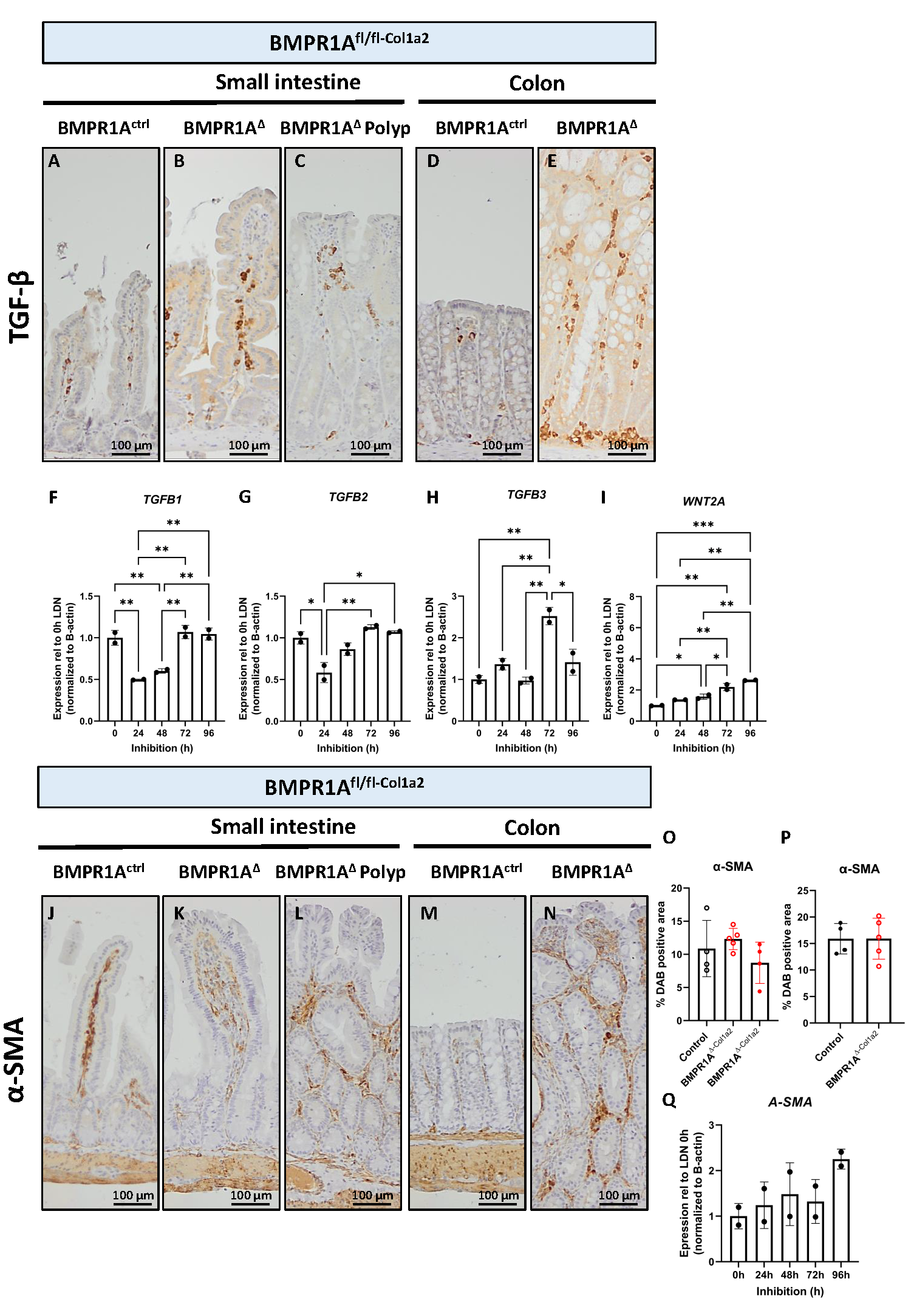

Supplement: Supplementary file 10 — Supplementary file10: Supplementary figure 6. Loss of BMPR1A signaling resulted in increased TGF-β expression. A-E) TGF-β was found to be increased in the intestine of BMPR1A∆-col1a2 mice compared to control mice. Although also increased in the small intestine, the increase was found to be only significant in the colon. F-I) Stimulation of CCD-18co fibroblasts with 200 nM LDN-193189 for up to 96 hours showed that fibroblasts alter their TGFβ1, TGFβ2 and TGFβ3 expression significantly when BMP signaling is inhibited. The expression of WNT2A increased gradually but significantly over time. J-P) No changes were observed in the percentage of α-SMA positive expression between BMPR1A∆-col1a2 mice and control mice. Q) Stimulation of CCD-18co with LDN-193189 also did not result in any significant changes of A-SMA expression. Bars represent mean ± SD. P <0.05 (*), <0.01(**) and <0.001(***) (TIF 2711 KB) [file 535_2022_1928_MOESM10_ESM.tif]

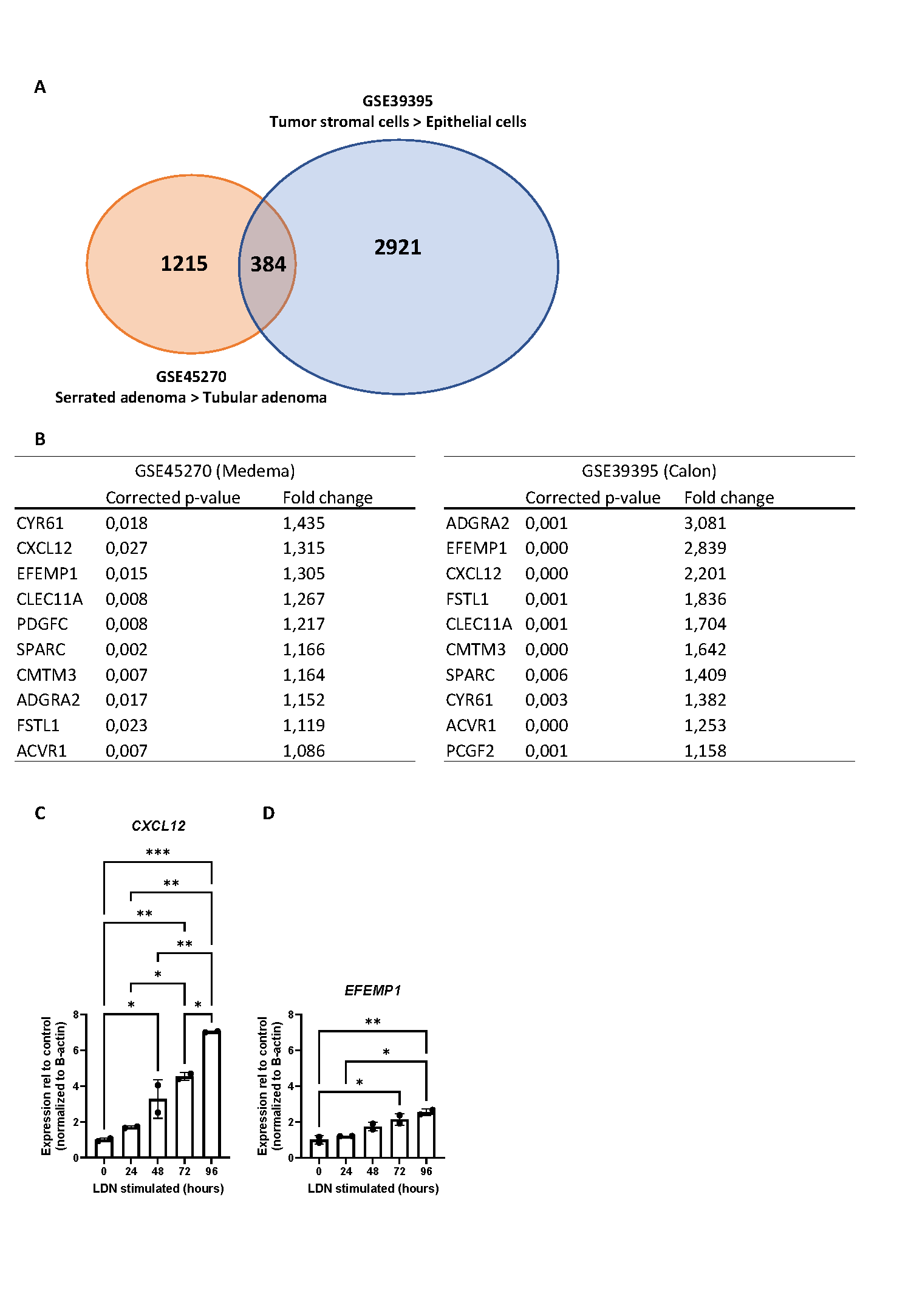

Supplement: Supplementary file 11 — Supplementary file11: Supplementary figure 7. CXCL12 is a factor specifically upregulated in serrated polyps and tumor stroma. A) The combination of two online publicly available data sets, GSE45270 and GSE39395, identified 384 differently expressed genes in both sets. From these 384 genes, 99 encoded for a secreted protein. B) From these 99 factors, 10 were expected to have an effect on epithelial cells. CXCL12 belonged to the top 3 most differentially expressed genes. C-D) Stimulation of CCD-18co fibroblasts for up to 96 hours with 200 nM LDN-193189 resulted in a significant increase of CXCL12 expression reaching almost 8-fold after 96 hours. EFEMP1 was also found to be significantly upregulated with a 2-fold increase in expression after 96 hours. Bars represent mean ± SD. P <0.05 (*), <0.01(**) and <0.001(***). (TIF 365 KB) [file 535_2022_1928_MOESM11_ESM.tif]

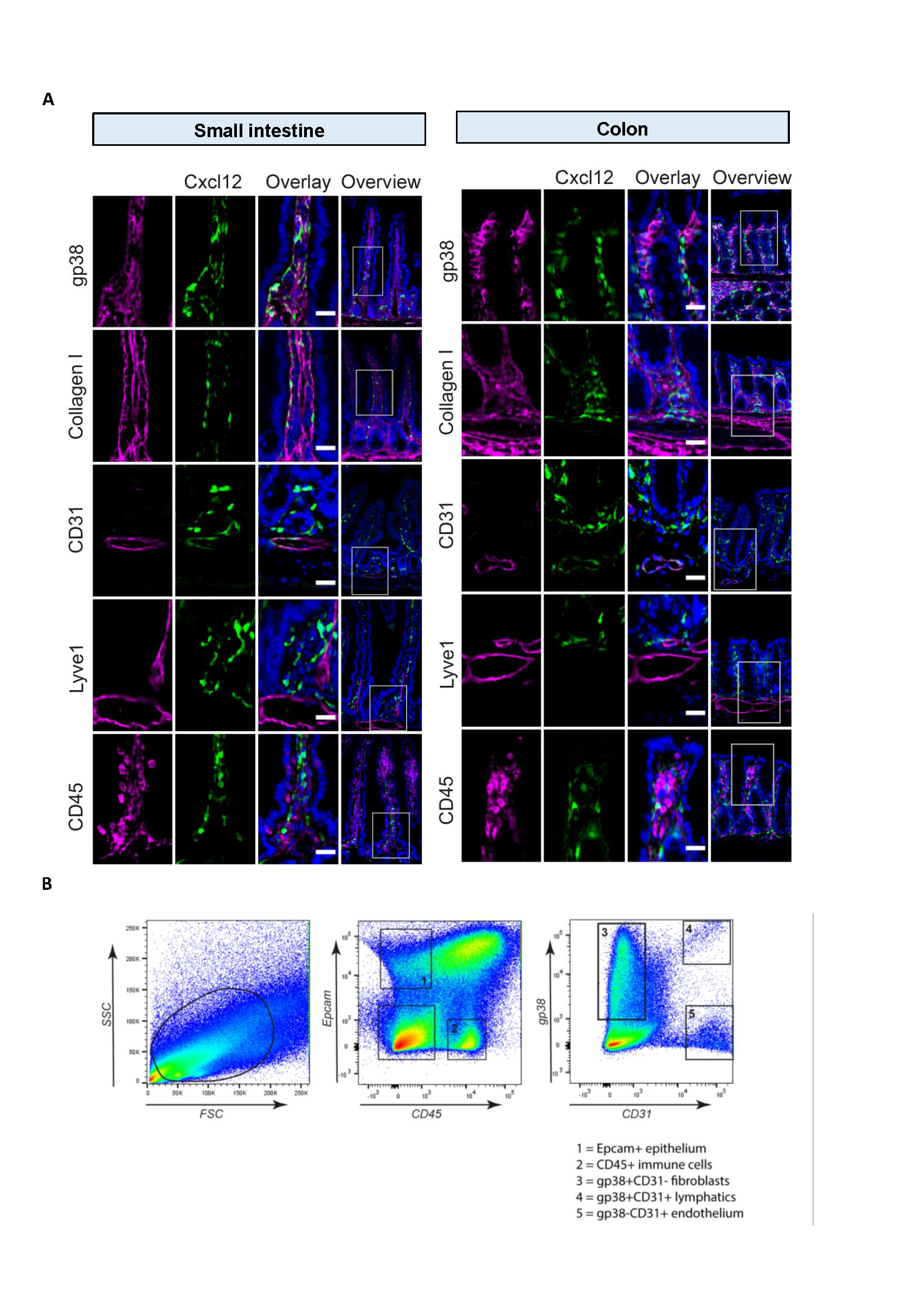

Supplement: Supplementary file 12 — Supplementary file12: Supplementary figure 8. CXCL12 expression is present in fibroblasts and endothelial cells but not immune cells and lymphatic endothelial cells. Immunofluorescent visualization of GFP in intestinal tissue from CXCL12-GFP mice showed that CXCL12-GFP was only present in the stroma of the small intestine and colon. A) CXCL12 expression co-localized with endothelial cells (CD31+) and fibroblasts (gp38+ and Collagen I+) but not with lymphatic endothelial cells (Lyve1+) and leukocytes (CD45+). B) FACS sort strategy for sorting epithelial cells, immune cells, fibroblasts, endothelial cells and lymphatic endothelial cells. (TIF 3840 KB) [file 535_2022_1928_MOESM12_ESM.tif]

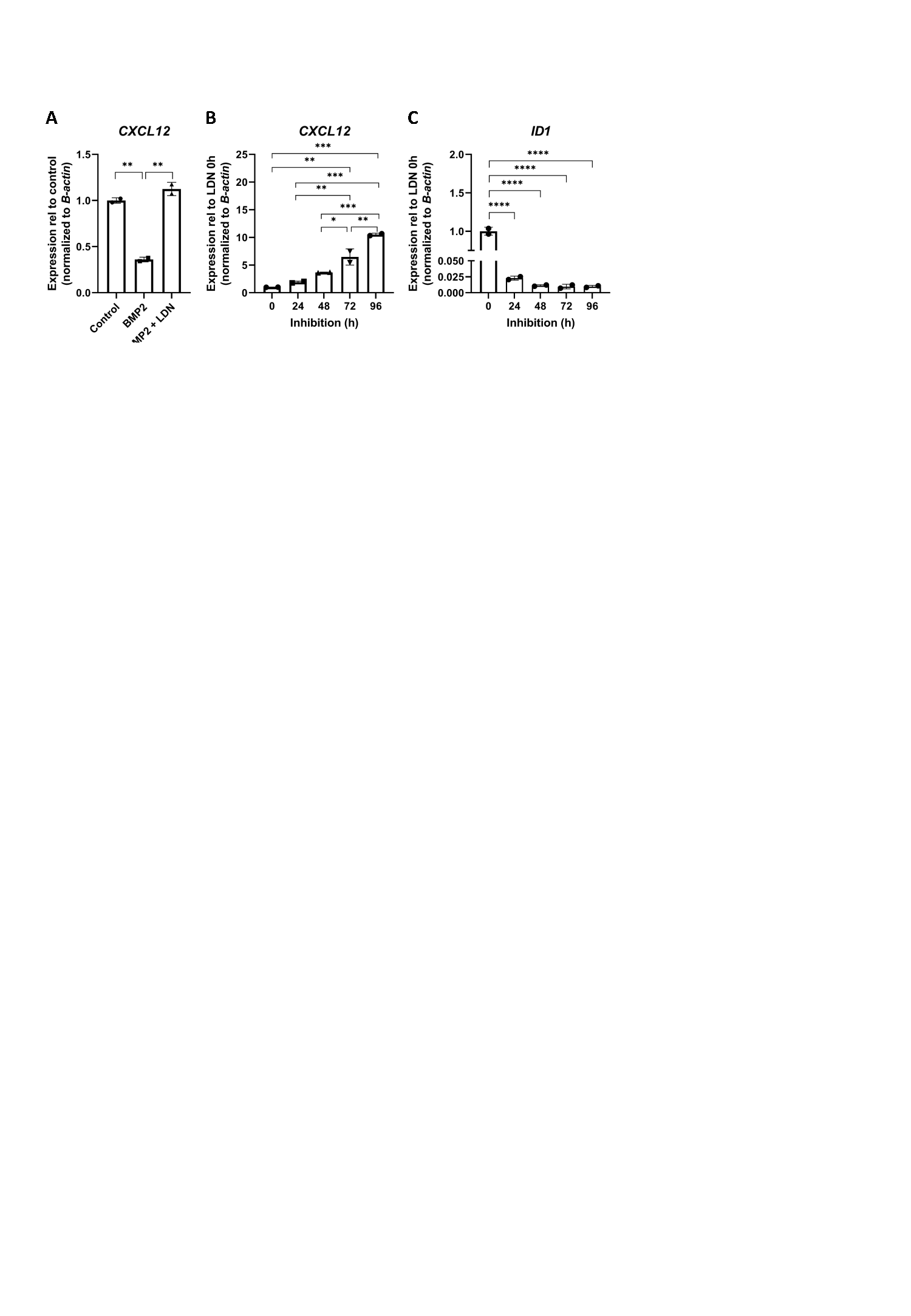

Supplement: Supplementary file 13 — Supplementary file13: Supplementary figure 9. BMP antagonists also regulate CXCL12 expression. A) Stimulation of 18co fibroblasts with 100 ng BMP2 for 24h resulted in a significant downregulation of CXCL12. Downregulation was prevented when 200 ng recombinant Noggin was present in the medium. B) Stimulating CCD-18co fibroblasts or primary colonic fibroblasts for up to 96h with Noggin showed a gradual increase of CXCL12 over time. C) ID1 expression also decreased, showing that BMP signaling was successfully prevented throughout the stimulation. Bars represent mean ± SD. P <0.05 (*), <0.01(**), <0.001(***) and <0.0001(****). (TIF 319 KB) [file 535_2022_1928_MOESM13_ESM.tif]

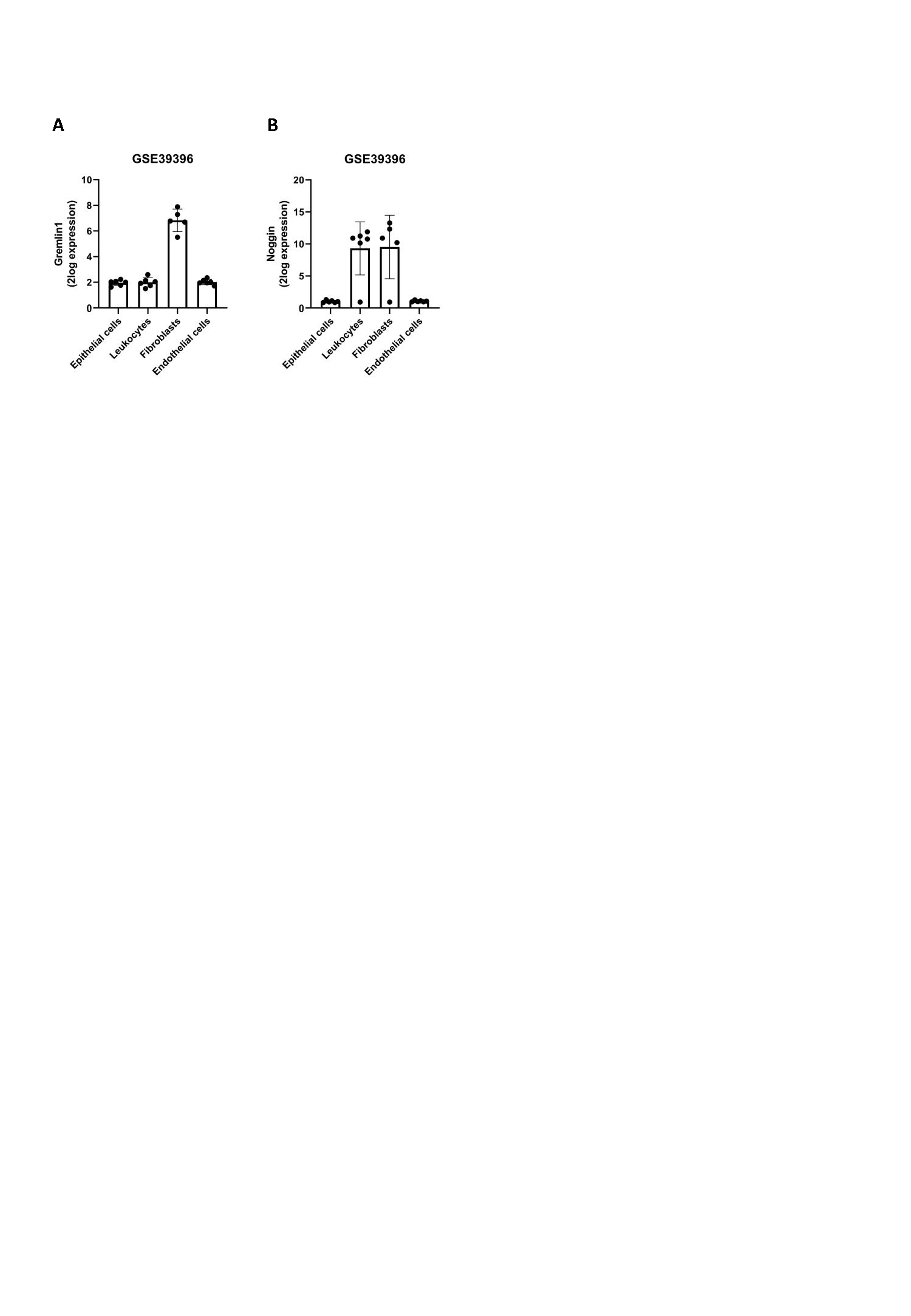

Supplement: Supplementary file 14 — Supplementary file14: Supplementary figure 10. Expression of GREMLIN1 and NOGGIN by different cell types in CRC. A) GREMLIN1 gene expression was higher in the fibroblast population compared with leukocytes, endothelial cells, and epithelial cells isolated by FACS (GSE39396). B) NOGGIN gene expression was found to be high in both fibroblasts and leukocytes compared with endothelial cells and epithelial cells. (TIF 338 KB) [file 535_2022_1928_MOESM14_ESM.tif]

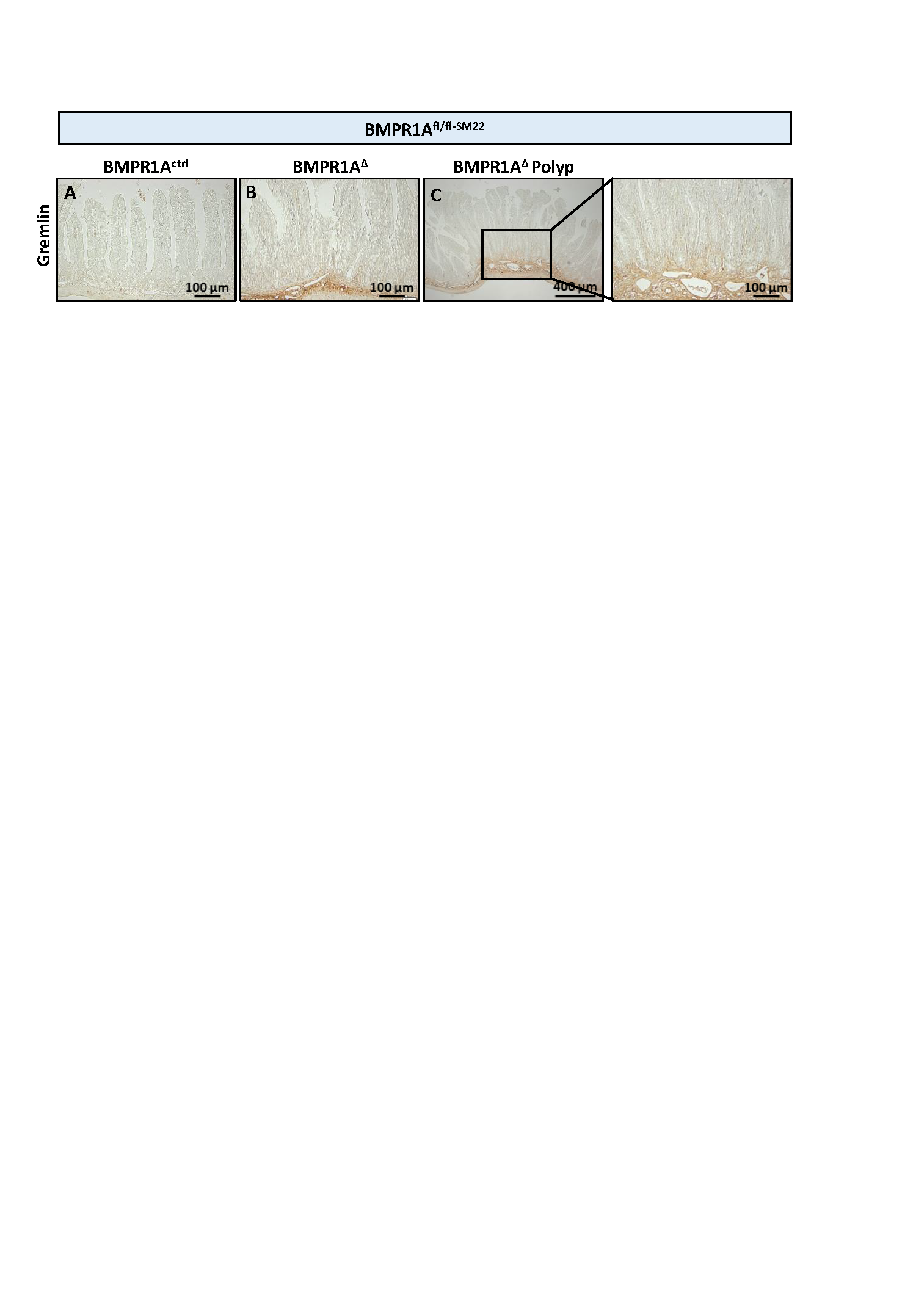

Supplement: Supplementary file 15 — Supplementary file15: Supplementary figure 11. Loss of BMPR1A signaling resulted in higher Gremlin expression in the intestines of BMPR1A∆-SM22 mice. A-D) A clear increase of Gremlin expression was observed in KO mice compared to controls as assessed by immunohistochemistry using an anti-GREM1 antibody. (TIF 865 KB) [file 535_2022_1928_MOESM15_ESM.tif]

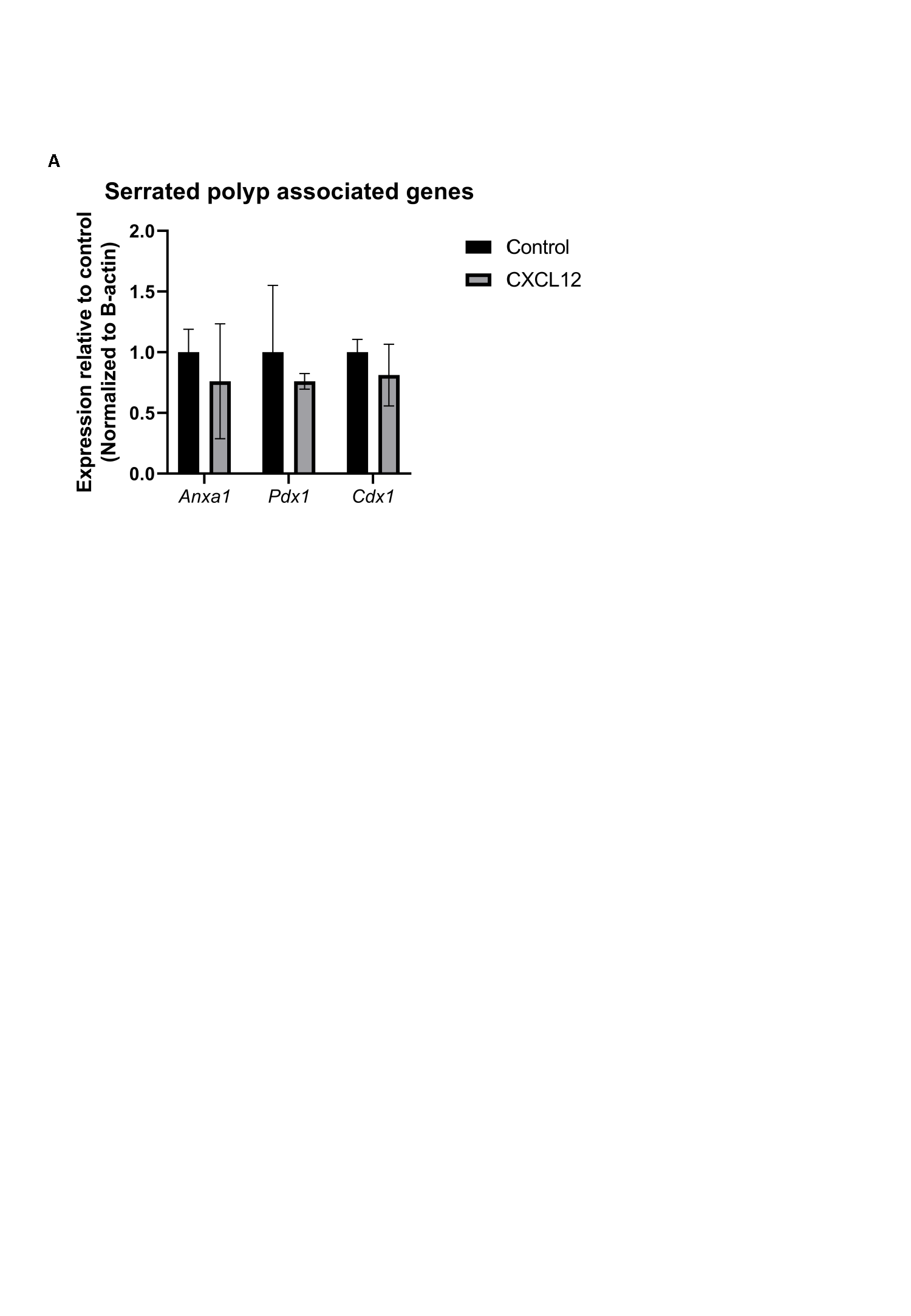

Supplement: Supplementary file 16 — Supplementary file16: Supplementary figure 12. Stimulation of intestinal organoids with CXCL12 does not lead to significant changes in expression of genes associated with the serrated phenotype. A) stimulation of intestinal organoids with CXCL12 did results in changes of expression of Pdx1, Anxa1 and Cdx1. (TIF 320 KB) [file 535_2022_1928_MOESM16_ESM.tif]

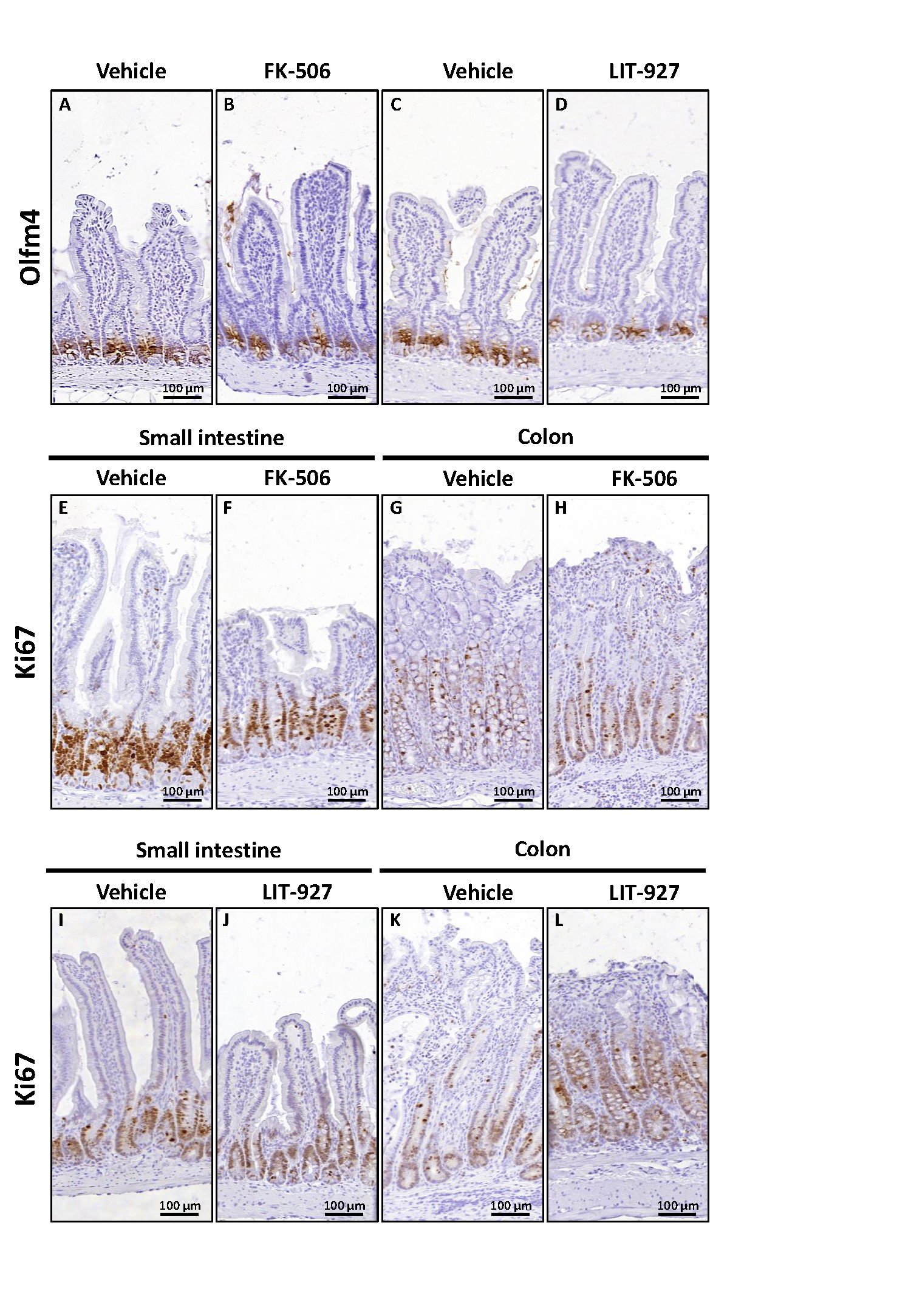

Supplement: Supplementary file 17 — Supplementary file17: Supplementary figure 13. Treatment of BMPR1A∆-Col1a2 mice with FK-506 leads to significant fewer olfm4 positive cells per crypt. A-L) Representative images of olfm4 and ki67 stainings taken from the mice treated with FK-506, LIT-927 or vehicle controls. (TIF 4510 KB) [file 535_2022_1928_MOESM17_ESM.tif]

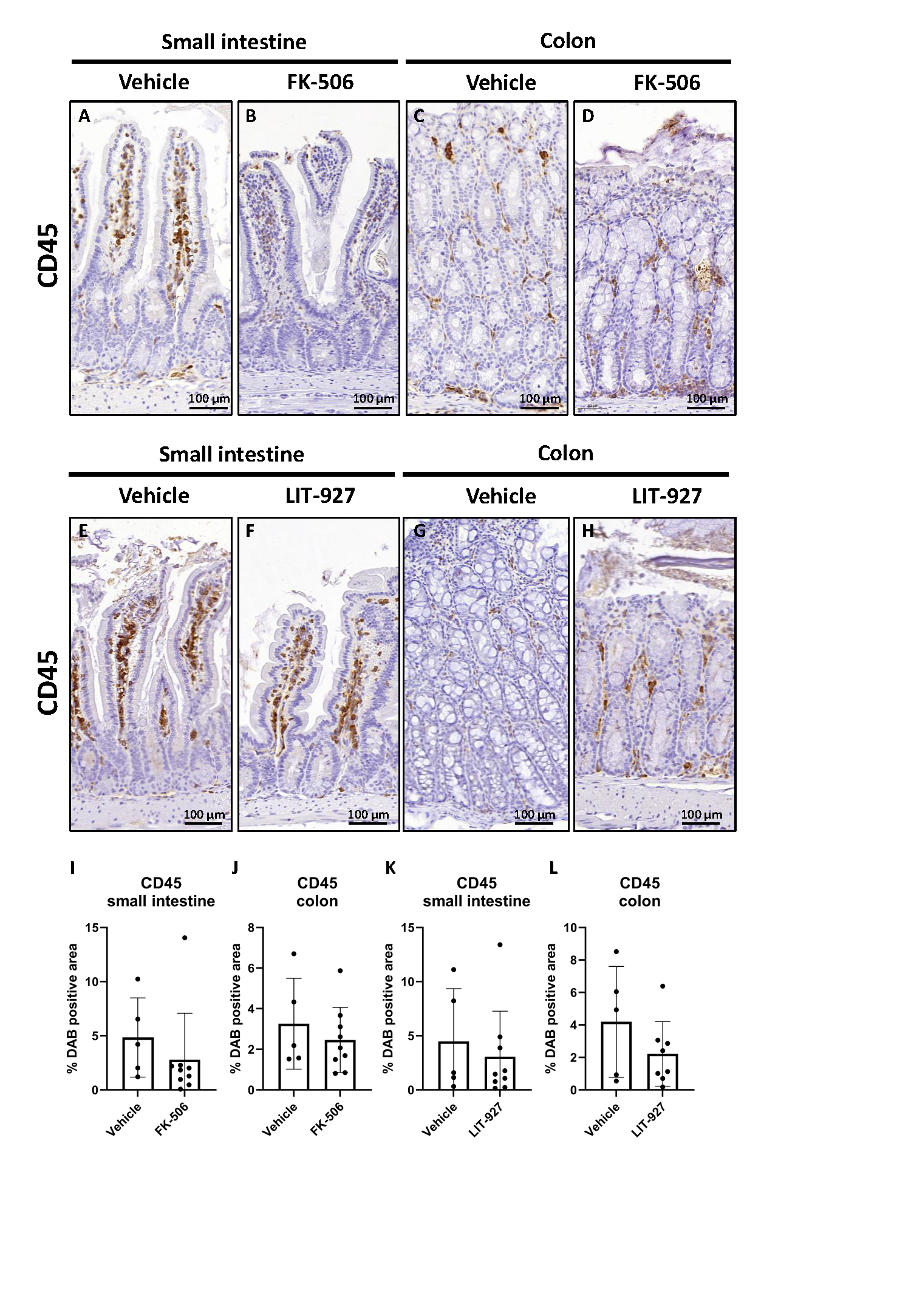

Supplement: Supplementary file 18 — Supplementary file18: Supplementary figure 14. BMPR1A∆-Col1a2 mice with FK-506 or LIT-927 does not lead to significant changes in the percentage of CD45 cells. A-L) While treatment of mice with FK-506 or LIT-927 resulted in a decrease of CD45 cells in the intestinal tissue, this decrease was found to be not significant. (TIF 3606 KB) [file 535_2022_1928_MOESM18_ESM.tif]

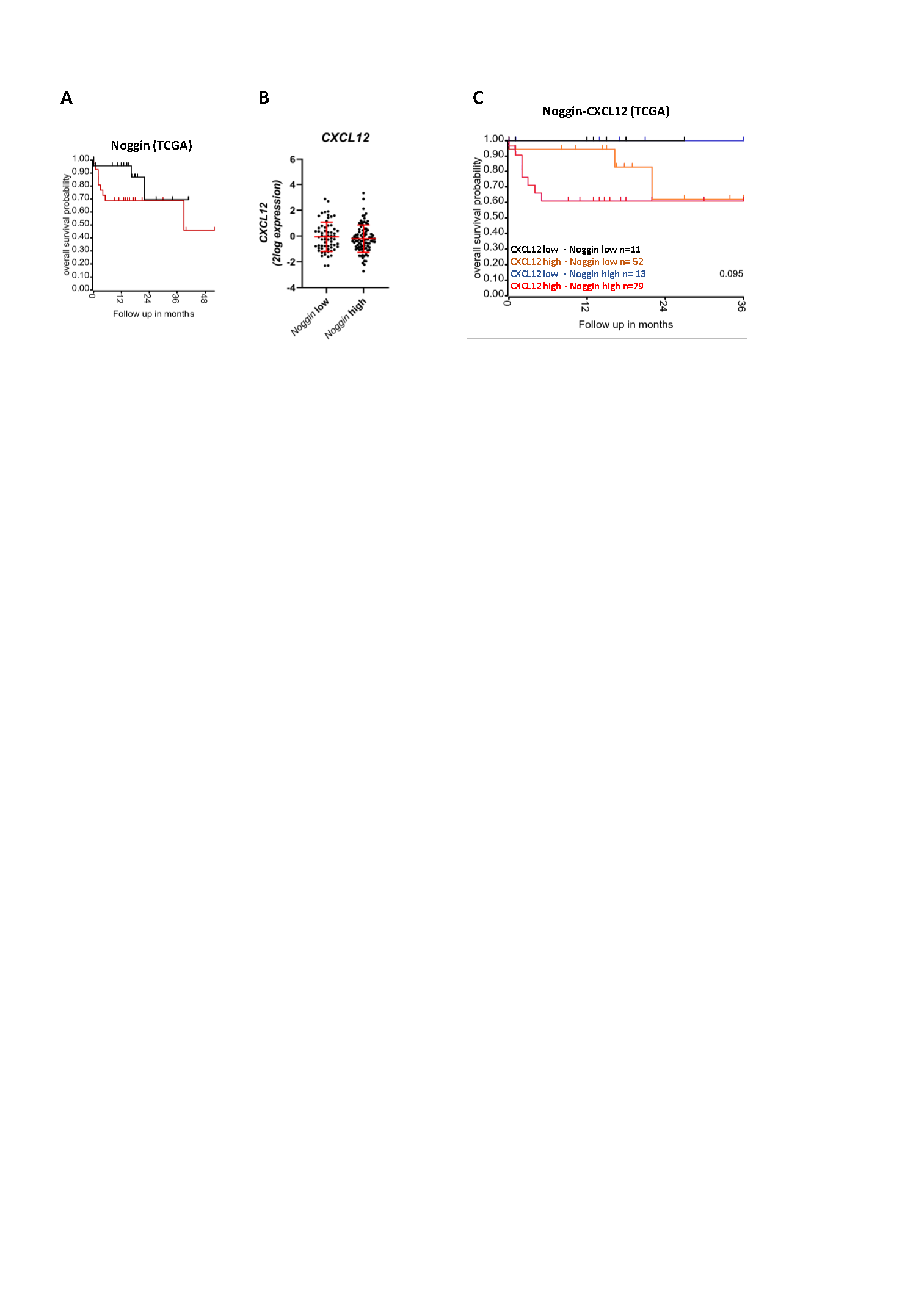

Supplement: Supplementary file 19 — Supplementary file19: Supplementary figure 15. NOGGIN expression is not significantly associated with patient survival. A) Stratification of NOGGIN in low and high expressing groups does not show a significant difference in patient survival between the groups. B) No association was found between NOGGIN expression and CXCL12 expression. C) Stratification of patients for NOGGIN and CXCL12 expression does not show a significant difference in patient survival between the groups. (TIF 326 KB) [file 535_2022_1928_MOESM19_ESM.tif]
